# EEGDecoder-x: an explainable deep learning framework for cross-subject EEG-based detection of Alzheimer's and Creutzfeldt–Jakob disease

**DOI:** 10.3389/fneur.2026.1851752

**Published:** 2026-07-20

**Authors:** Muhammad Suffian, Nadia Mammone, Cosimo Ieracitano, Giovanbattista Gaspare Tripodi, Angelo Pascarella, Edoardo Ferlazzo, Francesco Carlo Morabito

**Affiliations:** 1 DICEAM, Mediterranea University of Reggio Calabria, Reggio Calabria, Italy; 2 DICMaPI, University of Naples “Federico II”, Naples, Italy; 3 Neurology Unit, Great Metropolitan “Bianchi-Melacrino-Morelli” Hospital, Reggio Calabria, Italy; 4 Department of Medical and Surgical Sciences, Magna Græcia University of Catanzaro, Catanazaro, Italy; 5 Regional Epilepsy Centre, Great Metropolitan “Bianchi-Melacrino-Morelli” Hospital, Reggio Calabria, Italy

**Keywords:** Alzheimer's disease, brain-computer interface, Creutzfeldt-Jakob disease, cross-subject decoding, electroencephalography, explainable AI

## Abstract

Early detection of neurodegenerative diseases is critical. Distinguishing early-stage Creutzfeldt–Jakob disease (CJD) from “mimics” like Alzheimer's disease (AD) remains a major challenge; while EEG is valuable in advanced CJD, early-stage abnormalities are often non-specific and overlap with other rapidly progressive dementias. Deep learning offers promising EEG-based diagnostic solutions, but clinical adoption requires transparent decision-making, the interpretability of the features learned by deep learning models is equally important. In this context, careful model design and explainability are essential. In this paper, we propose a novel interpretable framework, *EEGDecoder-x*, for decoding EEG signals from subjects with Alzheimer's disease, Creutzfeldt–Jakob disease, and healthy controls, while providing insight into the model's learned characteristics. The EEGDecoder-x framework comprises two main components: a hybrid attention network for disease decoding (*EEGDecoder-Net*) and an explainability module (*EEGDecoder-XAI*). EEGDecoder-Net combines a convolutional neural network with a dual attention mechanism, followed by a classification layer, enabling efficient spatio-temporal feature extraction. EEGDecoder-XAI provides a comprehensive local and global explanations of the network's learning process for spatio-temporal dimensions. We validate the proposed framework using a Leave-One-Subject-Out evaluation paradigm, achieving *97.22% classification accuracy* on a dataset of 36 subjects (12 with AD, 12 with CJD, and 12 healthy controls), and outperforming the baseline models, demonstrating both the effectiveness and interpretability of EEGDecoder-x.

## 1 Introduction

Neurodegenerative diseases represent a growing global health challenge, driven by aging populations and the progressive, irreversible nature of these disorders. Among them, Alzheimer's disease (AD) is the most common cause of dementia, while Creutzfeldt–Jakob disease (CJD) is a rare but rapidly progressing condition characterized by severe cognitive decline. Despite their different pathological mechanisms and disease trajectories, early-stage symptoms of AD and CJD may sometimes overlap, making accurate and timely differential diagnosis particularly challenging (1–3).

Current diagnostic pipelines primarily rely on neuroimaging modalities and cerebrospinal fluid (CSF) biomarkers, which, although informative, are often expensive, invasive, and not readily scalable for large population screening. In contrast, electroencephalography (EEG) offers a promising alternative due to its non-invasive nature, relatively low cost, and high temporal resolution (4, 5). While EEG effectively identifies CJD in advanced stages through characteristic patterns, early-stage abnormalities are often non-specific and can overlap with other rapidly progressive dementias. EEG has been shown to capture disease-specific alterations in brain dynamics, making it suitable for computer-aided diagnosis of neurodegenerative conditions. However, translating EEG-based methods into real-world clinical applications remains challenging.

One key issue lies in the discrepancy between controlled research datasets and real clinical EEG recordings. In practical hospital settings, EEG signals are frequently contaminated by noise, motion artifacts, electrode disconnections, and abrupt signal discontinuities (6). These artifacts introduce non-stationarity and can significantly degrade the performance and reliability of machine learning models if not properly addressed. Furthermore, many existing studies overlook such imperfections by relying on curated datasets, limiting the robustness and clinical applicability of proposed methods.

Another critical limitation in the current literature is related to evaluation methodology. A large number of EEG-based classification studies employ trial-level cross-validation, where segments from the same subject may appear in both training and testing sets. This leads to data leakage, inflated performance metrics, and poor generalization to unseen subjects (7). To address this issue, subject-independent validation strategies such as Leave-One-Subject-Out (LOSO) cross-validation have been advocated as a more realistic benchmark (8, 9). Nevertheless, achieving high performance under LOSO settings is inherently difficult due to substantial inter-subject variability, especially in small and heterogeneous clinical datasets.

Beyond classification performance, interpretability has emerged as a fundamental requirement for deploying machine learning models in clinical environments. Clinicians require not only accurate predictions but also insights into the underlying decision-making process of the model. This is particularly important in EEG analysis, where understanding spatio-temporal patterns associated with neurological disorders can provide valuable neurophysiological insights. However, many deep learning approaches for EEG classification operate as black-box models, limiting their trustworthiness and clinical adoption. The existing works which explain or interpret the learning of EEG models lacks clear interpretability goals like explaining the EEG data on spatial, temporal, local, and global levels aligned with clinical protocols (10, 11).

To address these challenges, we propose EEGDecoder-x, a novel interpretable framework for cross-subject EEG-based classification of neurodegenerative diseases. The framework is specifically designed to balance three critical aspects: (i) robustness to real-world EEG artifacts, (ii) reliable subject-independent evaluation, and (iii) model interpretability. EEGDecoder-x consists of two main components: EEGDecoder-Net, a hybrid deep learning architecture that integrates one dimensional convolutional neural networks with dual attention mechanisms (channel attention and transformer) to effectively capture both local and global spatio-temporal dependencies, and EEGDecoder-XAI, an explainability module that provides multi-level insights into the learned representations.

The proposed framework is evaluated on a clinical EEG dataset comprising AD, CJD, and healthy control (CNTRL) subjects, where artifact-contaminated segments are explicitly identified and excluded based on expert annotations. A LOSO cross-validation strategy is employed to ensure subject-independent evaluation. Despite the inherent challenges of cross-subject variability, the proposed approach achieves a classification accuracy of 97.22%, demonstrating strong generalization capability.

The main contributions of this work are summarized as follows:

1. We propose a novel explainable EEG-based framework (EEGDecoder-x) for the classification of neurodegenerative diseases under realistic clinical conditions.
2. We design a compact hybrid CNN–Attention architecture that effectively captures spatio-temporal EEG patterns while maintaining computational efficiency with a comprehensive explainability module that provides clinically meaningful insights into model behavior for spatio-temporal relevance.
3. We adopt a rigorous LOSO evaluation protocol to ensure subject-independent validation and reliable performance assessment.
4. We contribute to open source code to promote reproducibility and Open Science, available at GitHub.^1^

## 2 Background

### 2.1 Conventional feature extraction techniques

EEG-based analysis has shown considerable promise in identifying alterations in brain dynamics associated with neurodegenerative disorders. Earlier approaches predominantly focused on handcrafted features, such as spectral characteristics and functional connectivity measures, which were subsequently classified using traditional machine learning techniques (12). Mammone et al. (13) proposed the permutation disalignment index (PDI) as a quantitative measure for distinguishing mild cognitive impairment (MCI) from AD using EEG recordings. Their study revealed increased PDI values in the delta and theta frequency bands among MCI patients who later progressed to AD, suggesting that EEG alterations may precede the manifestation of clinical symptoms. In a subsequent study, Mammone et al. (14) introduced the permutation Jaccard distance (PJD) to assess longitudinal changes in brain connectivity. Their findings indicated that MCI patients who converted to AD exhibited a noticeable rise in PJD values within the delta and theta bands, whereas non-converting patients did not show similar trends. Amezquita-Sanchez et al. (15) developed an automated framework for the diagnosis of MCI and AD by leveraging advanced signal processing techniques, specifically combining Multiple Signal Classification (MUSIC) with Empirical Wavelet Transform (EWT) for feature extraction. Their method achieved a classification accuracy of approximately 90%, demonstrating the effectiveness of combining sophisticated signal processing with machine learning for EEG-based diagnosis.

### 2.2 Machine learning and neural network approaches

Machine learning approaches have also been widely explored for EEG-based diagnosis of neurodegenerative disorders. These methods typically rely on handcrafted features derived from time, frequency, or time-frequency domains, which are then used with classifiers such as support vector machines (SVM), k-nearest neighbors (k-NN), or random forests. While such approaches have demonstrated reasonable performance, their effectiveness is often limited by the quality and representativeness of manually engineered features. In recent years, deep learning techniques, particularly convolutional neural networks (CNNs), have gained significant attention due to their ability to automatically learn hierarchical and discriminative representations directly from raw EEG signals (8, 16, 17). Morabito et al. (18) proposed an autoencoder-based framework to distinguish early-stage CJD from other forms of rapidly progressive dementia (RPD). By fine-tuning a globally pre-trained model, their method achieved an average accuracy of approximately 89% in separating CJD from RPD, with similar performance reported for distinguishing CJD from AD and CNTRL subjects. EEGNet (16) was a good breakthrough for EEG decoding with CNNs, it is a compact CNN using depthwise and separable convolutions to efficiently learn spatial and temporal EEG patterns with very few parameters. Temporal Convolutional Network (TCN) (19) was proposed with a fully convolutional sequence model using causals and dilated 1D convolutions with residual connections to capture long-range temporal dependencies without recurrence. Another CNN-based model Deep ConvNet (8) was proposed with deep CNN and multiple convolution-pooling blocks that progressively learn hierarchical temporal and spatial features directly from raw EEG signals.

More recently, Transformer-based architectures have shown strong potential in modeling long-range temporal dependencies in sequential data (20). ADformer (21) was introduced as an end-to-end framework that leverages a spatio-temporal Transformer to capture multi-scale spatial and temporal features from raw EEG signals. The model was evaluated across multiple datasets and achieved a maximum F1-score of 92.89%. A Convolutional Transformer Network (CTNet) (22) is a hybrid architecture combining convolutional layers for local EEG feature extraction with Transformer encoders to model global temporal dependencies via self-attention, this model have achieved good performance for motor imagey tasks. EEGConformer (23) is another hybrid model which integrates CNN-based spatial-temporal feature extraction with Conformer blocks (self-attention + convolution) to jointly learn local and global EEG representations.

Despite these advancements, many existing studies rely on evaluation protocols and experimental settings that do not accurately reflect real-world clinical scenarios, thereby limiting their robustness and generalizability in practical applications.

### 2.3 Explainable AI approaches for neurodegenerative disease diagnosis

Recent studies have increasingly focused on incorporating explainability into EEG-based diagnostic frameworks to enhance clinical interpretability and trustworthiness. Lopes et al. (24) proposed a hybrid framework combining CNNs with saliency map analysis for AD diagnosis. The saliency maps were generated from validation data to highlight the most informative regions contributing to the model's decisions. The features extracted from these regions were used to train a support vector machine (SVM) classifier. The study provided channel-level importance analysis derived from the identified salient regions. In another study proposed by Morabito et al. (25), an explainable deep learning framework was introduced to investigate the conversion from MCI to AD using high-density EEG (HD-EEG) recordings. The method utilized channel-by-frequency representations as input to a CNN-based classification model. For interpretability, Gradient-weighted Class Activation Mapping (Grad-CAM) was applied to generate visual explanations over channel-frequency maps, highlighting the most relevant spectral-spatial features influencing the model's predictions. Bunterngchit et al. (26) proposed a hybrid temporal convolutional framework combined with disease-specific attention mechanisms to learn multi-scale spatio-temporal representations under a LOSO evaluation setting. The interpretability of the model is supported by gradient-based saliency maps, integrated gradients, and perturbation-based occlusion analysis. Another study by Mouazen et al. (10) employed an autoencoder-based architecture to learn compact EEG representations, where features extracted from the decoder embeddings were subsequently fed into a bidirectional LSTM for classification. For explainability, SHapley Additive exPlanations (SHAP) were utilized to quantify the contribution of different frequency band features. Li et al. (11) proposed an Interpretable CNN (InterpretableCNN) to identify EEG biomarkers associated with AD across multiple cognitive task conditions. CAM was employed to generate spatio-temporal visual explanations, producing topographic maps that highlight the most discriminative EEG regions over time. Although recent studies have incorporated explainable AI methods for EEG-based analysis, many approaches do not explicitly define interpretability objectives in a clinically meaningful manner. Specifically, they often fail to provide comprehensive, multi-level explanations spanning spatial, temporal, local, and global perspectives, which are essential for alignment with established clinical interpretation protocols.

## 3 Materials and methods

**Figure 1:**
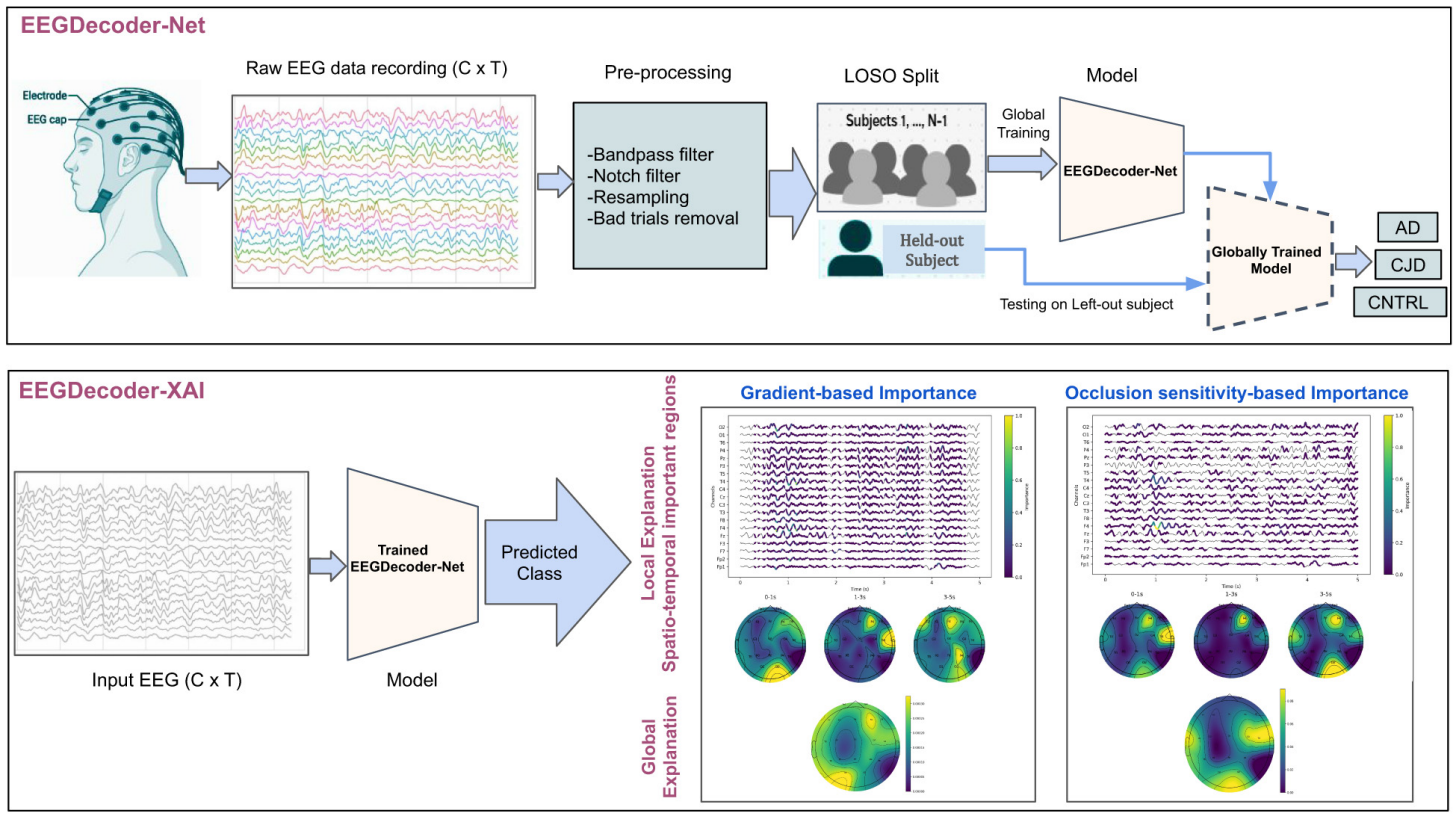
Architecture of the EEGDecoder-x framework. **(Top)** EEG data acquisition and pre-processing are performed, followed by partitioning into Leave-One-Subject-Out (LOSO) splits, then the model is trained on *N*−1 subjects and evaluated on the held-out subject. **(Bottom)** the explainability workflow of EEGDecoder-XAI is illustrated, in which the trained model is used to make predictions, and the predictions are subsequently interpreted at both local and global levels using gradient-based and occlusion-based saliency maps.

In this section, a general architecture of EEGDecoder-x is shown in Figure 1. The dataset used is described in section 3.1, data pre-processing is described in section 3.2, the time-frequency analysis was conducted to visualize the data across the subjects and it is described in section 3.3. The learning model EEGDecoder-Net is described in section 3.4, the explainability method for the learning model called EEGDecoder-XAI is described in section 3.5, and in the last the experimental setup is described in section 3.6 including the details of baseline models adopted for comparison with the proposed model. Figure 1 (top) shows the process of EEG data acquisition, pre-processing, partitioning of data into LOSO splits, training of the EEGDecoder-Net model on *N*−1 subjects, and evaluation on the held-out subject. Figure 1 (bottom) shows the explainability workflow calling it EEGDecoder-XAI, in which the globally trained model is used to predict the disease class, and the predictions are subsequently interpreted at both local and global levels using gradient-based and occlusion-based saliency maps.

### 3.1 Dataset

A total of 36 participants were enrolled in this study at the Regional Epilepsy Center and Unit of Neurology of the Great Metropolitan “Bianchi-Melacrino-Morelli" Hospital in Reggio Calabria. The cohort was stratified into three groups (*n* = 12 per group): patients with AD, patients with CJD, and healthy controls. Clinical diagnoses were established by expert neurologists according to the revised international consensus criteria. For AD, participants were evaluated following the current diagnostic criteria and recommendations (27, 28). For CJD, diagnoses were confirmed according to the updated diagnostic criteria for sporadic CJD (29), incorporating clinical findings, EEG patterns, and biomarker evidence. The control group consisted of age-matched individuals with no history of neurological disorders and unremarkable clinical and instrumental findings. Clinical and demographic characteristics of the study population are reported in Table 1. Patients with AD had a significantly higher age compared to CJD patients and CNTRL (79 vs. 61 vs. 61 years, respectively; *p* = 0.001), while sex distribution did not differ significantly across groups. Disease duration at the time of EEG recording was 16 weeks (median, range 1–28 weeks) in the CJD group and 72 weeks (median, range 20–480 weeks) in the AD group.

**Table 1:** Demographic and clinical characteristics of the study population.

Characteristic	CJD (*n* = 12)	AD (*n* = 12)	CNTRL (*n* = 12)	*p*-value
Sex: female/male, n (%)	7/5 (58.3/41.7)	5/7 (41.7/58.3)	7/5 (58.3/41.7)	0.640
Age: median (IQR), years	61 (52.5–70)	79 (67–84.5)	61 (52–67)	0.001
Disease duration at EEG time, median (range), weeks	16 (1–28)	72 (20–480)	–	–

Bold indicates a statistically significant difference (*p* < 0.05).

IQR, interquartile range.

It is to be noted that all data were anonymized prior to analysis in accordance with data protection requirements, the dataset and associated documentation are available at GitHub.^2^ EEG signals were recorded using 19 electrodes positioned according to the 10–20 international system, with Cz serving as the ground electrode. The recordings were originally acquired under clinical conditions, resulting in variations in recording duration across subjects depending on diagnosis and clinical protocol. The EEG signals were sampled at a rate of 256 Hz (resampled for one subject from 512 to 256 Hz), band-pass filtered between 1.6 and 40 Hz, notch filtered at 50 Hz to suppress power-line interference.

### 3.2 Data pre-processing

**Figure 2:**
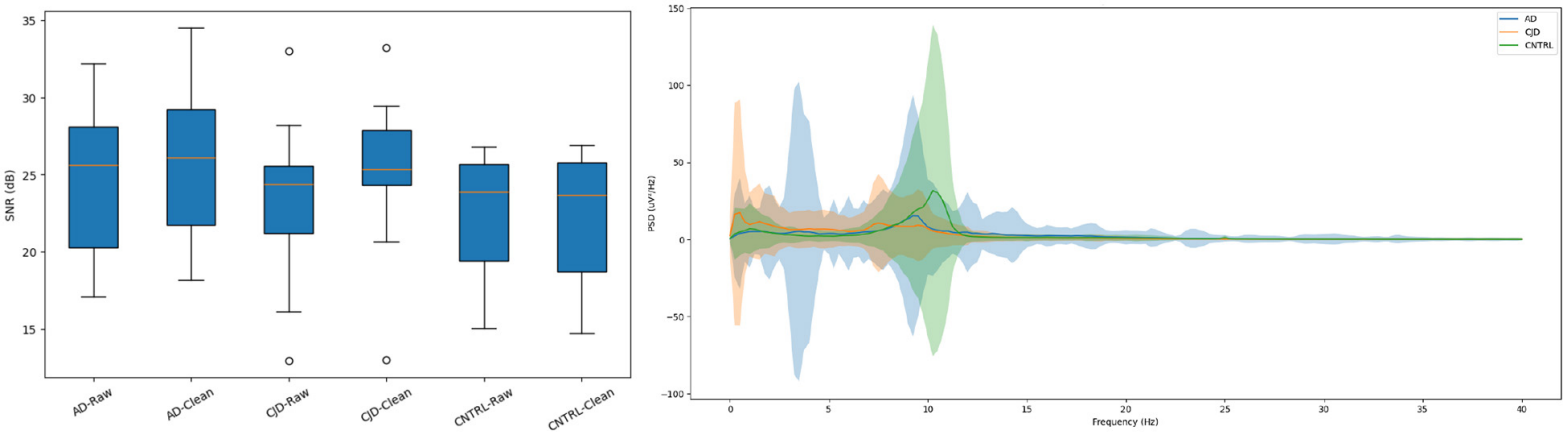
Signal quality and spectral analysis across classes. **(Left)** Comparison of SNR between raw and clean EEG trials. **(Right)** PSD distributions for AD, CJD, and CNTRL subjects, shown with mean and standard deviation, highlighting class-specific spectral characteristics.

We applied Independent Component Analysis (ICA) to clean the EEG signal and isolate non-brain artifacts, such as eye blinks and muscle activity. These artifactual components were then subtracted from the data to produce a clean signal while preserving the underlying neural activity. ICA was applied independently to subjects at the initial phase of pre-processing and the cleaned EEG signals were saved. The EEG signals were segmented into non-overlapping 5-s trials. Although the total recording duration varied across subjects, an equal number of trials (40 trials) were extracted for each subject to ensure class balance across AD, CJD, and CNTRL groups. All subjects retained at least 40 clean trials except for one CJD subject, which contained only 17 clean trials. To avoid introducing artificial patterns or bias, we did not perform augmentation or synthetic trial generation for this subject. The resulting data was organized into LOSO splits and used for subsequent model training and evaluation. To assess signal quality, the signal-to-noise ratio (SNR) was computed for both raw and clean EEG trials across all subject groups. As illustrated in Figure 2 (left), clean trials consistently exhibit higher SNR values compared to raw trials, confirming the effectiveness of the pre-processing pipeline in suppressing noise and non-neural artifacts while preserving physiologically relevant brain activity. In addition, power spectral density (PSD) analysis was performed and shown in Figure 2 (right) to examine the distribution of spectral power across frequency bands for AD, CJD, and CNTRL subjects. The PSD plots provide complementary insights into class-specific spectral characteristics, with observable variations in band power distributions across groups. These findings further support the presence of disease-related alterations in neural oscillatory activity and validate the use of cleaned EEG signals for reliable downstream analysis.

### 3.3 Time-frequency analysis of EEG across classes

**Figure 3:**
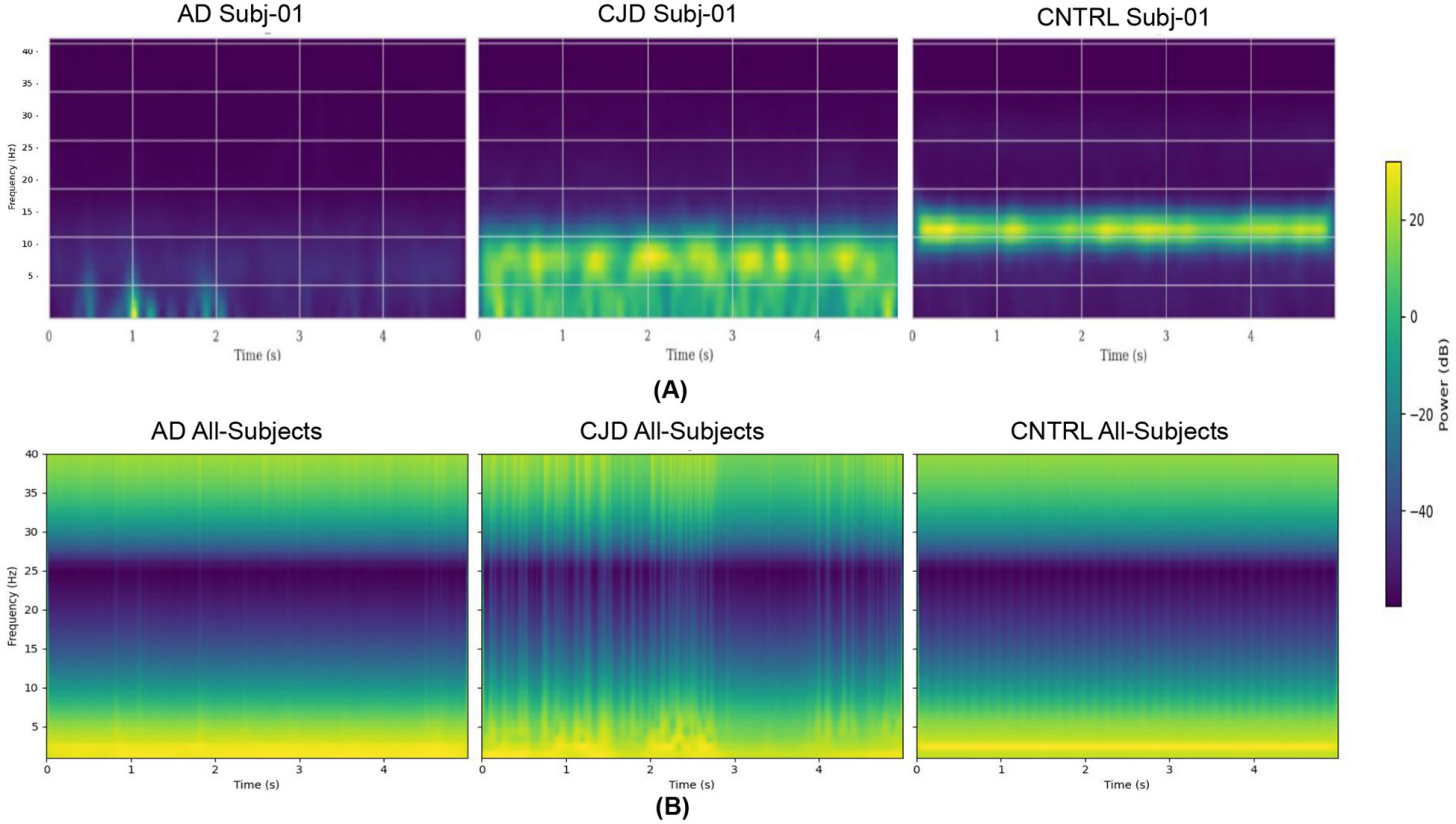
CWT-based Time-Frequency maps. **(A)** shows TF-maps for individual subjects, and **(B)** shows averaged TF-maps across all trials and subjects.

EEG signal dynamics across all subject groups were analyzed in the time-frequency (TF) domain using the Continuous Wavelet Transform (CWT). For each pre-processed EEG trial, the CWT was applied to obtain a joint time–frequency representation, enabling the characterization of non-stationary neural oscillations that are known to be altered in neurodegenerative conditions. The resulting TF maps were utilized for qualitative assessment of spectral-temporal patterns across AD, CJD, and CNTRL subjects. Representative TF maps from three subjects per class are shown in Figure 3A, illustrating subject-specific EEG dynamics. Figure 3B presents class-wise averaged TF maps across all subjects.

From a neurophysiological perspective, distinct alterations in oscillatory activity can be observed. In particular, CJD subjects exhibit more pronounced transient and broadband activity in the TF domain at the individual level, reflecting the rapid and diffuse cortical dysfunction associated with diseases. In contrast, AD subjects tend to show a relative shift toward lower-frequency activity (e.g., delta and theta bands), consistent with cortical slowing reported in the literature. However, when averaged across subjects, these differences become less prominent due to inter-subject variability, although subtle distinctions in spectral power distribution remain observable.

### 3.4 EEGDecoder-Net: learning model

**Figure 4:**
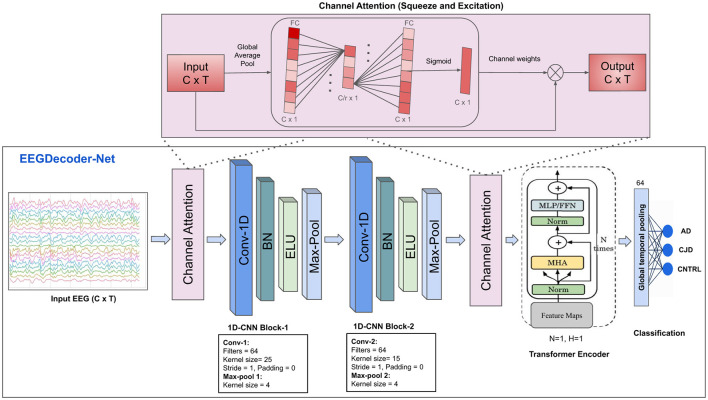
The EEGDecoder-Net architecture. The input EEG signal is first processed by a channel attention module, followed by two 1-D convolutional feature extraction blocks. The resulting feature maps are refined using a second channel attention module and passed to a transformer encoder for temporal modeling. A final classification layer produces predictions for AD, CJD, and CNTRL.

The proposed model, EEGDecoder-Net, consists of a hybrid CNN-Transformer with channel attention mechanism for EEG classification. The model architecture diagram is shown in Figure 4.

Given an input EEG segment X∈ℝ^*C*×*T*^, where *C* = 19 denotes the number of channels and *T* = 1, 280 corresponds to the temporal samples (5 s at 256 Hz). The input EEG is first processed by a channel attention module that learns the relative importance of input channels using a squeeze-and-excitation mechanism. The channel attention is shown at the top of Figure 4. The reweighted signal by channel attention is then passed through two 1D convolutional blocks, each consisting of convolution, batch normalization, ELU activation, and max-pooling, to extract temporal features. A second channel attention module is applied to the learned feature maps to emphasize informative representations. Subsequently, the features are reshaped and fed into a Transformer encoder with multi-head self-attention to capture long-range temporal dependencies. The output is globally averaged over time and passed through a fully connected layer for classification into AD, CJD, and CNTRL classes. The different components of the EEGDecoder-Net are described in the following subsequent sections.

#### 3.4.1 Channel attention mechanism

To enhance interpretability and emphasize informative EEG channels, a channel-wise attention mechanism is employed based on the squeeze-and-excitation (SE) principle. The channel attention via squeeze-and-excitation adaptively reweights EEG channels by modeling inter-channel dependencies, enabling the network to emphasize clinically relevant brain regions while suppressing less informative or noisy channels. In squeeze, global average pooling is applied along the temporal dimension: $z_{c}=\frac{1}{T}\overset{T}{\underset{t=1}{\sum }}X_{c,t},where\;c=1,\ldots ,C$, thus *C*×*T* becomes *C*×1. In excitation, the channel descriptor z∈ℝ^*C*^ is passed through a bottleneck multi-layer perceptron: s = σ(W_2_δ(W_1_z)) where ${\text{W}}_{1}\in {\mathbb{R}}^{\frac{C}{r}\times C}$, ${\text{W}}_{2}\in {\mathbb{R}}^{C\times \frac{C}{r}}$, δ(·) denotes the ELU activation, σ(·) is the sigmoid function, and *r* is the reduction ratio. The reduction value *r* = 4 is used for input attention and *r* = 8 for feature attention, ratio *r* for input attention is determined based on the number of EEG input channels which are 19 and for feature attention based on the extracted features with CNN. Although the original SE formulation recommends (*r* = 16) which was suboptimal for EEG due to the small number of input channels (in our setup), leading to excessive compression. The recalibration of attention weights s are used to rescale the input: ${\tilde{X}}_{c,t}=s_{c}\cdot X_{c,t}$ This attention module is applied both at the input level (to weight EEG channels) and after CNN feature extraction (to weight learned feature maps). Overall, *r* = 4 and *r* = 8 provide optimal trade-off between representational capacity and compression at both input-level and feature-level attention stages in the proposed architecture.

#### 3.4.2 1D-CNN for feature extraction

The recalibrated signal is passed through two temporal convolutional blocks to extract local temporal patterns from multichannel EEG signals. The first convolutional layer uses *n*_in_ = 19 channels, *n*_out_ = 64 channels with kernel size *k* = 25, followed by batch normalization, ELU activation, and max-pooling (pool = 4). The second convolutional layer has 64 channels with a kernel size 15, batch normalization, ELU, and max-pooling (pool = 4). These layers progressively reduce the temporal dimension while increasing feature abstraction. After the second pooling layer, the feature maps have size 64 × 75 (channels × temporal steps) and are permuted to sequences of shape 75 × 64 for the Transformer encoder.

A second channel attention module is applied to recalibrate learned feature maps for feature-level attention weights.

#### 3.4.3 Transformer encoder for temporal modeling

The Transformer encoder models long-range temporal dependencies in EEG signals. The encoder consists of one layer of a multi-head self-attention (MHA) with one head, and a position-wise feedforward network (FFN). The output of MHA is passed through a feedforward network with residual connections and layer normalization applied after both the attention and feedforward blocks to stabilize training.

#### 3.4.4 Temporal pooling and classification

The Transformer outputs are aggregated along the temporal dimension using global average pooling producing a fixed-size embedding vector p∈ℝ^64^. This embedding is fed into a fully connected layer for three-class classification (AD, CJD, CNTRL).

### 3.5 EEGDecoder-XAI: explainability

To interpret the proposed EEGDecoder-Net model, we introduce *EEGDecoder-XAI*, a *post-hoc* explainability framework that provides spatio-temporal attribution of model predictions. The framework integrates mainly two approaches: gradient-based saliency and occlusion-based saliency, along with attention to identify discriminative spatio-temporal regions and EEG channels contributing to classification decisions, serving local and global explanations.

#### 3.5.1 Gradient-based importance

Given an input EEG segment X∈ℝ^*C*×*T*^ and the predicted class score *y*_*k*_, we compute gradient-based saliency with respect to the input: $\text{G}=\frac{\partial y_{k}}{\partial \text{X}}$, where G∈ℝ^*C*×*T*^ captures the sensitivity of the prediction to each channel-time point. To obtain a robust temporal importance map, we aggregate gradients across channels. This produces a temporal saliency highlighting discriminative time regions.

**Channel importance from attention mechanisms**. In parallel, we extract channel-wise attention weights from both input-level and feature-level attention modules. These weights quantify the relative importance of EEG channels, derived from learned squeeze-and-excitation operations. Additionally, Transformer self-attention encodes temporal dependencies across the sequence.

**Local explanation: spatio-temporal attribution**. To obtain a unified spatio-temporal local explanation, we combine gradient-based temporal importance with channel attention weights. This way we get S∈ℝ^*C*×*T*^ which represents the joint importance of channel *c* at time *t*. The spatio-temporal saliency map S is visualized for the corresponding EEG signal/segment, and it is highlighted using a color map (e.g., dark purple-to-yellow), emphasizing important time regions in highly weighted channels. For the sake of better visibility, we highlighted the top-5 channels at important times. This formulation enables localization of salient EEG activity in both spatial (channels) and temporal domains.

**Global explanation: visualization with topographic mapping**. The spatio-temporal saliency maps S of all EEG segments are averaged, and then channel-wise importance is obtained by temporal aggregation. These values are projected onto a scalp layout to visualize spatial distributions of neural activity at the global level, to identify the most important channel for a specific disease/class.

#### 3.5.2 Occlusion-based attribution

To further enrich the explainability mechanism and to validate gradient-based explanations, we employ an occlusion-based method that perturbs the input and measures its effect on model predictions. For each channel *c* and temporal window [*t, t*+*w*], we occlude the signal (e.g., set to the mean value) and compute the change in prediction score. Δ*y*_*c, t*_ = *y*_*k*_(X)−*y*_*k*_(X_\(*c, t*)_), where X_\(*c, t*)_ denotes the input with the specified region occluded. By sliding the window across time, we obtain an occlusion-based importance map O_*c, t*_ = Δ*y*_*c, t*_ This map provides an alternative estimate of spatio-temporal relevance.

**Local explanation**. Since the occlusion-based importance map O_*c, t*_ holds joint importance of channel *c* at time *t* in the same shape as S for gradient-based importance, it is visualized in the same way as for local explanation in gradient-based importance. For the sake of better visibility, we highlighted the top-5 channels at important times.

**Global explanation**. The global explanations are produced by aggregating the temporal dimension for all trials and visualized with topographical plots. The multi-perspective approach of EEGDecoder-XAI enables robust and physiologically meaningful interpretation of EEG-based disease classification.

### 3.6 Experimental setup

Hyperparameters were selected through an empirical tuning process, considering both classification performance and computational efficiency. For the CNN feature extractor, kernel sizes and pooling configurations were systematically adjusted to balance temporal resolution and model complexity. The final design comprises two 1D convolutional layers with kernel sizes of 25 and 15, respectively, each followed by batch normalization, ELU activation, and max-pooling with a pooling factor of 4, providing an effective trade-off between representational capacity and efficiency. The Transformer encoder was configured with one encoder layer and one attention head to model long-range temporal dependencies while keeping the architecture lightweight. It is worth mentioning that increasing the number of encoder layers and attention heads did not result in marginal performance gains, hence we kept the model light-weight instead of increased computational cost and energy consumption.

The model was trained using the Adam optimizer with a learning rate of 1 × 10^−3^ and weight decay of 1 × 10^−4^. A batch size of 64 was used, with training conducted for up to 30 epochs. Early stopping was applied to prevent overfitting and reduce unnecessary computational overhead.

We focused on the generalizability of the model by training on data from multiple subjects *N*−1 and evaluating on the *N*^*th*^ unseen subject. The cross-subject protocol (LOSO) was following: In each iteration of LOSO, one subject is held out for testing, and the remaining *N*−1 subjects are used for model training. The data from the *N*−1 subjects are split into five folds, and fivefold cross-validation is used for training and validation. Early stopping is applied based on validation performance. The model with the best validation performance across folds is selected and saved. The best saved model is employed to test with the held-out subject's data. The test performances of the model are reported in the results.

For fair comparison, we consider established EEG decoding baselines, including EEGNet (EEGNet-8,2) (16), Deep ConvNet (8), TCN (19), CTNet (22), and EEGConformer (23). All models are configured using the same input time window and number of EEG channels as the proposed framework to ensure a consistent and comparable evaluation setting. All the baseline models are imported from Braindecode.^3^ which is an open-source Python toolbox (8).

All experiments were implemented using the PyTorch deep learning framework. Model training was conducted on a workstation running Ubuntu, equipped with an NVIDIA RTX 4000 Ada Generation GPU, an Intel Xeon(R) CPU @ 2.30 GHz, and 128 GB of RAM.

## 4 Results

### 4.1 Model performance results

The performance of the proposed model and baseline models were evaluated using standard metrics, namely: Accuracy, Precision, Recall, F1-score, and Cohen Kappa.

The models were evaluated using a LOSO approach. Subject-level classification was obtained by aggregating trial-level predictions for each subject. Specifically, we applied a majority voting scheme, where each subject was assigned the class that appeared most frequently among its predicted trial labels. A subject is considered correctly classified if the majority-voted label matches the ground truth label of that subject. For example during testing, out of 40 trials the majority predicted label was considered to assign the class of that label, subject-level Accuracy, Precision, Recall, and F1-score were then averaged across all subjects and were reported in Table 1.

As reported in Table 2, the experimental results demonstrate that the proposed *EEGDecoder-Net* outperforms the baseline models across all metrics, confirming its strong overall classification performance. In particular, *EEGDecoder-Net* achieved an average accuracy, precision, recall, F1-score, and specificity of 97.22%, 97.16%, 97.07%, 97.01%, and 95.55%, respectively. In contrast, the baseline models showed lower performance across all metrics.

**Table 2:** Model performance on LOSO.

Model	Accuracy	Precision	Recall	F1-score	Cohen Kappa	CI (95%)
EEGNet	94.00	88.00	85.00	86.00	88.00	4.35
EEGConformer	94.70	94.88	94.75	94.67	92.06	4.20
Deep ConvNet	93.29	94.00	93.37	93.28	89.94	4.75
CTNet	94.07	94.38	94.15	94.00	91.10	4.55
TCN	93.36	93.51	93.41	93.30	90.05	4.20
EEGDecoder-Net (proposed)	97.22	97.16	97.07	97.01	95.55	2.75

Evaluation metrics are averaged across all subjects, where each subject performance was computed from the total trials of that subject.

Further, to observe the trial-level performance of models, the confusion matrix for each held-out subject was recorded, and the sum of all confusion matrices was aggregated to obtain a final confusion matrix to observe the total trials correctly classified by the model.

**Figure 5:**
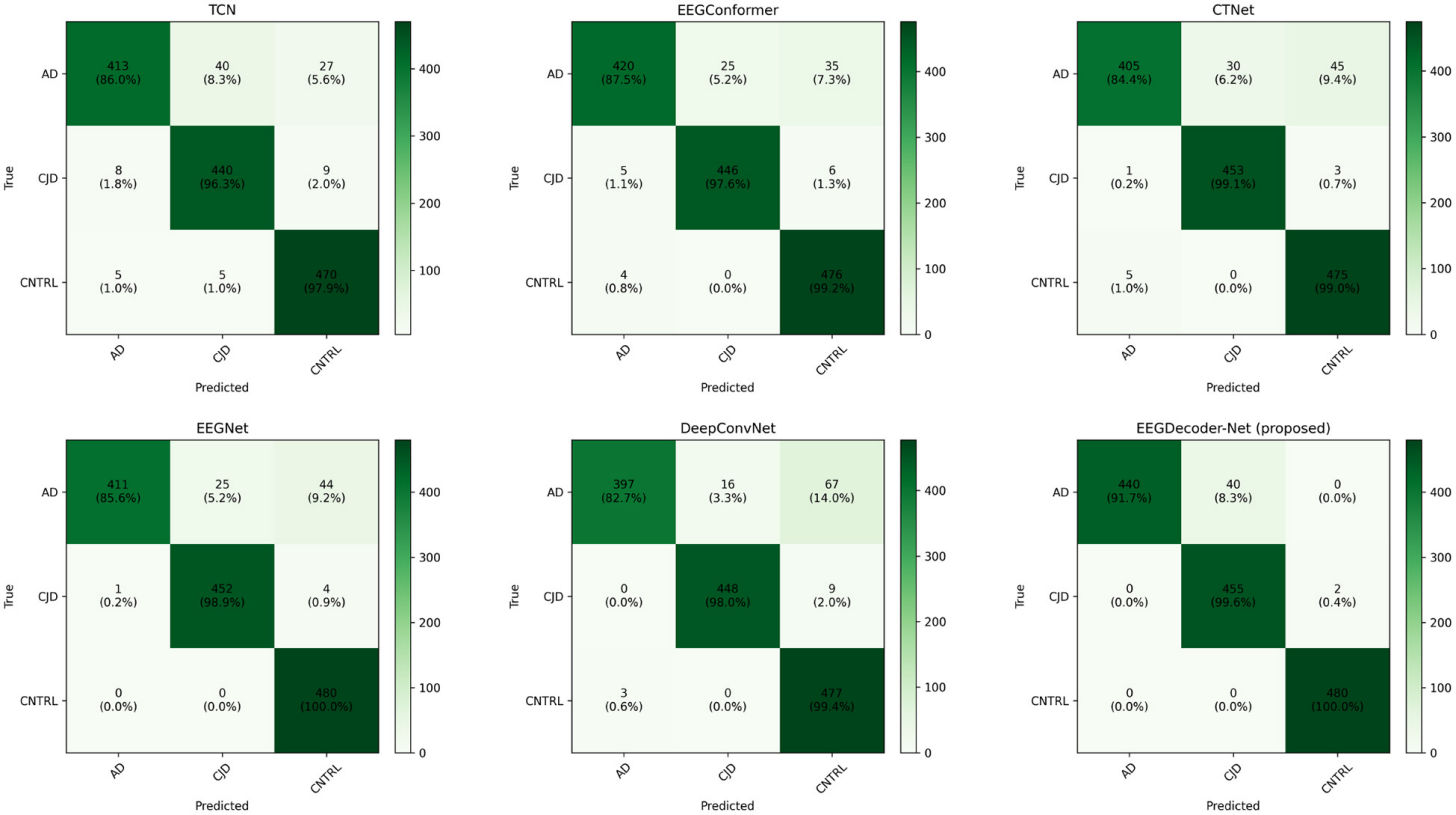
Confusion matrices produced by TCN, EEGConformer, CTNet, EEGNet, DeepConvNet, and EEGDecoder-Net in the AD vs. CJD vs. CNTRL classification using the LOSO approach. The confusion matrices show aggregated trials across all subjects (percentage of trials correctly classified for each class).

Figure 5 presents the final confusion matrices of *EEGDecoder-Net* and the baseline models. The matrices show consistently strong classification performance across all three classes. Compared to the baseline models, *EEGDecoder-Net* demonstrates superior performance, with low false-positive and false-negative rates, confirming its robust overall classification capability.

To assess whether performance differences among models were statistically significant, subject-level results obtained from the LOSO evaluation were analyzed using non-parametric repeated-measures tests. A Friedman test revealed a significant effect of model architecture on classification performance (χ^2^ = 23.49, *p* = 2.72 × 10^−4^), indicating significant differences among the evaluated models. *Post-hoc* pairwise comparisons were performed using Wilcoxon signed-rank tests between the proposed EEGDecoder-Net and each baseline. The proposed model significantly outperformed DeepConvNet (*p* = 0.0115) and CTNet (*p* = 0.0115). However, differences relative to EEGNet (*p* = 0.0803), EEGConformer (*p* = 0.4615), and TCN (*p* = 0.6547) did not reach statistical significance. These results demonstrate that the proposed architecture achieves statistically significant improvements over several convolution-based baselines while maintaining performance comparable to the strongest competing methods. It is also important to note that a substantial proportion of subjects were classified perfectly by multiple models, resulting in a ceiling effect that reduces the magnitude of pairwise differences despite high overall performance. Consequently, statistical comparisons were performed at the subject level rather than relying solely on aggregate performance metrics.

**Figure 6:**
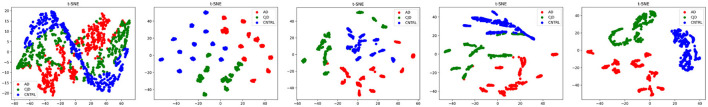
Visualization of learned feature maps/embeddings extracted from different model layers shown with t-SNE. (**Left** to **Right**) Input channel attention layer, Conv1-layer, Conv2-layer, Mid-level channel attention layer, and final layer.

To further analyze feature separability, t-SNE visualization was employed. Figure 6 shows the t-SNE plots of feature embeddings extracted from different layers of the models: from left to right, the input channel attention layer, Conv1 layer, Conv2 layer, mid-level channel attention layer, and the final layer. The plots demonstrate progressively improved class-wise clustering, with the final layer exhibiting the clearest separation between classes, highlighting the strong discriminative capability of the proposed model.

In addition to performance, we compared the model complexity and memory requirements of *EEGDecoder-Net* with several baseline models, as summarized in Table 3. While *EEGDecoder-Net* has a higher number of parameters than lightweight models such as EEGNet (374.67K vs. 6.92K) and TCN (41.22K), it remains substantially smaller than EEGConformer (969.06K) and comparable to CTNet and DeepConvNet. The total model size of *EEGDecoder-Net* is 4.47 MB, striking a balance between model capacity and memory efficiency, which supports its strong performance without excessive computational cost.

**Table 3:** Model complexity and memory comparison.

Metric	EEGConformer	TCN	EEGNet	CTNet	DeepConvNet	EEGDecoder-Net
Total params (K)	969.06	41.22	6.92	184.73	282.03	374.67
Trainable params (K)	969.06	41.22	6.92	184.73	282.03	374.67
Total size (MB)	14.11	2.61	3.65	7.78	2.08	4.47

#### 4.1.1 Ablation study

The ablation study is conducted to evaluate the contribution of each architectural component in the proposed EEGDecoder-Net model and the results are presented in Table 4. The full model integrates input attention, convolutional feature extraction, feature attention, and Transformer-based temporal modeling, achieving the best overall performance with a relatively compact parameter size of 375.5K parameters. To further analyze the influence of Transformer depth and complexity, variant A1 increases the Transformer configuration to two layers and two attention heads. Although this substantially increases the parameter count to 655.8K, the improvement in accuracy is not significant, indicating that deeper Transformer configurations costed only increased computational complexity. The impact of channel attention mechanisms is investigated in variants A2 and A3. Removing the input attention module (A2) slightly reduces the model capacity and degrades performance, suggesting that early-stage channel-wise attention helps suppress irrelevant EEG patterns before feature extraction. Similarly, removing the feature attention module (A3) also decreases performance, highlighting the importance of adaptive feature recalibration after CNN processing. Variant A4 removes the Transformer encoder while retaining the CNN and attention modules. The considerable reduction in parameters to 93.5K demonstrates the lightweight nature of the CNN-only backbone; however, the expected performance degradation confirms that Transformer-based global dependency modeling plays a critical role in capturing long-range temporal relationships in EEG signals. Variant A5 excludes the CNN feature extractor while preserving the Transformer and attention modules. Although the parameter count remains relatively high (283.7K), the absence of convolutional layers weakens local feature learning, indicating that CNN layers are essential for extracting discriminative low-level EEG representations before Transformer processing. Variant A6 uses only CNN layers, achieves lower performance despite having the smallest parameter count (92.4K), indicating insufficient global contextual modeling. In contrast, the Transformer-only model (A7) maintains a large parameter count (364.5K) but lacks efficient local feature extraction, limiting classification capability. Variant A8 combines CNN and Transformer modules without channel attention mechanisms, showing improved performance over single-module variants but still underperforming the full model. Overall, the ablation results confirm that each component contributes positively to the final performance, while the complete architecture achieves the best trade-off between model complexity and classification accuracy.

**Table 4:** Ablation study of the proposed CNN-Transformer EEG model.

Model variant	Input Attn	CNN	Feature Attn	Transformer	Params (K)	Accuracy (%)
Full model	✓	✓	✓	✓	375.5	97.22
A1 (2L, 2H)	✓	✓	✓	✓	655.8	97.30
A2	×	✓	✓	✓	374.6	96.10
A3	✓	✓	×	✓	373.6	95.90
A4	✓	✓	✓	×	93.5	91.45
A5	✓	×	✓	✓	283.7	89.60
A6	×	✓	×	×	92.4	90.35
A7	×	×	×	✓	364.5	88.60
A8	×	✓	×	✓	373.6	94.55

✓ indicates the component is enabled, while × indicates the component is removed. Parameter counts are reported in thousands (K).

### 4.2 Explainability results

In this section, the explanation results are presented according to the computation of local and global explanations for both gradient-based and occlusion-based strategies as described in section 3.5.

**Figure 7:**
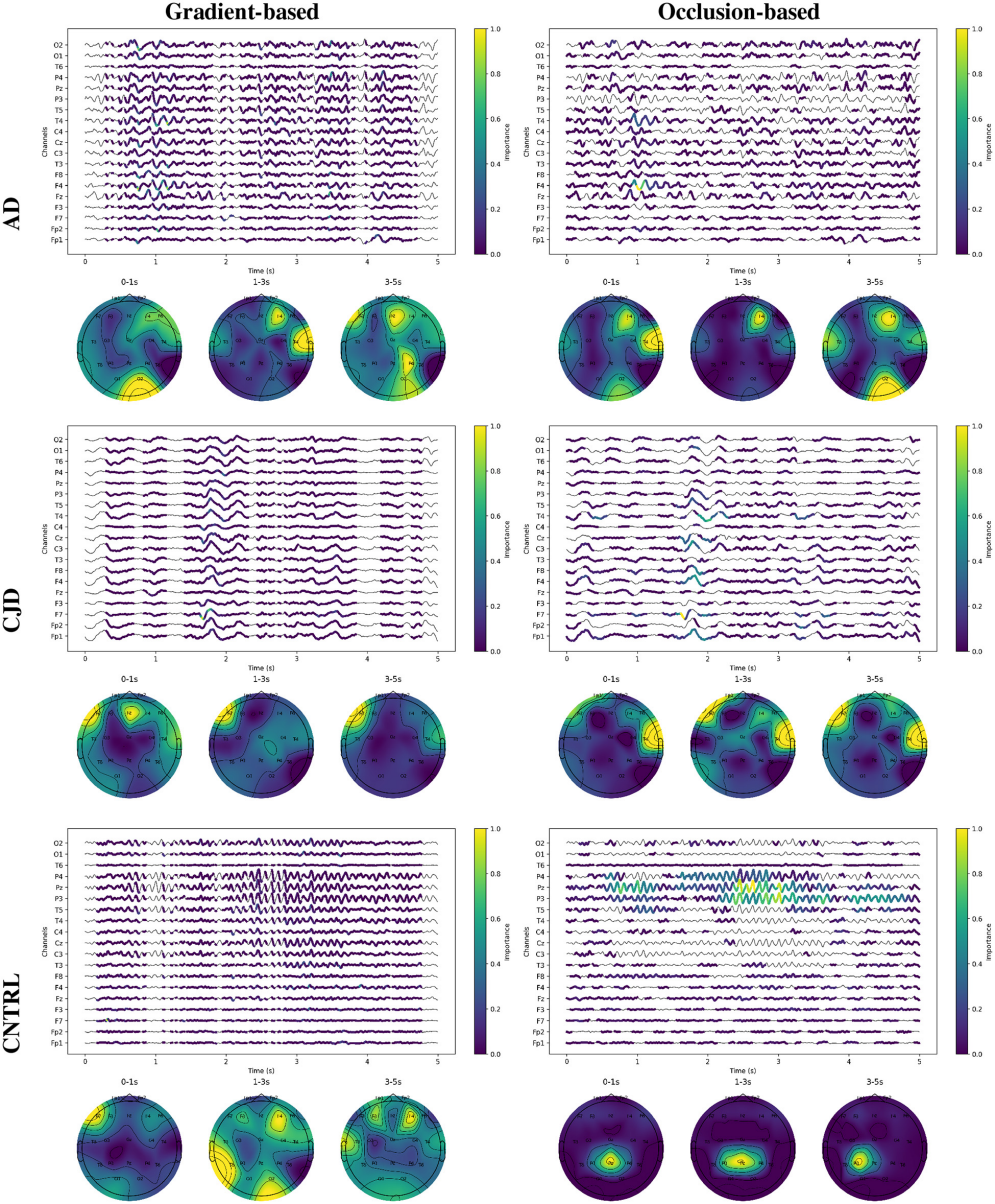
Local explanations are shown with a topographical map. The rows in the plot shows to classes (AD, CJD, and CNTRL) and columns shows specific explanations strategy for these classes (gradient-based and occlusion-based explanations).

Figure 7 shows representative local explanations for one EEG trial from a single subject in each class (AD, CJD, and CNTRL). The rows in the plot shows to classes (AD, CJD, and CNTRL) and columns shows specific explanations strategy for these classes (gradient-based and occlusion-based explanations). For each class, the input trial (19 × 1,280) is highlighted over the time of 5 s with spatio-temporal importance as a saliency map S∈ℝ^*C*×*T*^ which represents the joint importance of channel *c* at time *t* (top), and a corresponding topomap at different time intervals shows the topographical activity (bottom). In the saliency map S, each channel shows its importance/relevance toward the final outcome of the model and the importance is shown with a color bar (from low to high). Similarly, the topographical maps are plotted for three different time points over the 5 s to show channel importance. The first topomap shows the average importance from 0 to 1 s, second topomap shows importance from 1 to 3 s, and the third topomap shows importance from 3 to 5 s. For each class, the saliency map and topographical maps are plotted from the corresponding explanation method such as gradient-based and occlusion-based, and presented in parallel to observe the agreement in spatio-temporal importance.

The gradient-based explanations highlight the most salient channel-time regions by combining temporal gradients with channel attention weights, enabling precise localization of discriminative neural activity. In contrast, the occlusion-based approach identifies important regions by perturbing overlapping temporal segments, resulting in a denser distribution of salient regions along the time axis. It can be observed from the class AD that saliency map with gradient-based explanations shows important time regions around 1 s, and between 3 and 4 s, and its corresponding topomap shows F4, F8, and O2; F4 and T4; and F7, Fz, F4, P4, and O2 as important channels, respectively, mainly frontal and occipital regions. This behavior of gradient-based local explanation is agreed by the occlusion-based local explanation, which also highlight the same temporal regions around 1 s and in between 3 and 4 s. Further, it shows F4 and O2 as continuously important channels over the time, mainly frontal and occipital regions. Similarly, explanations for CJD both gradient-based and occlusion-based agree on the frontal region and partially on and temporal region. However, there is a slight disagreement between both explanation strategies for CNTRL class, to observe the extent of agreement and disagreement across all trials and subjects we have performed an agreement analysis between both explanation strategies and described in subsequent section. Notably, consistent activation patterns can be observed across both explanation methods, where several channels are commonly identified as important, providing mutual validation of the explanations.

**Figure 8:**
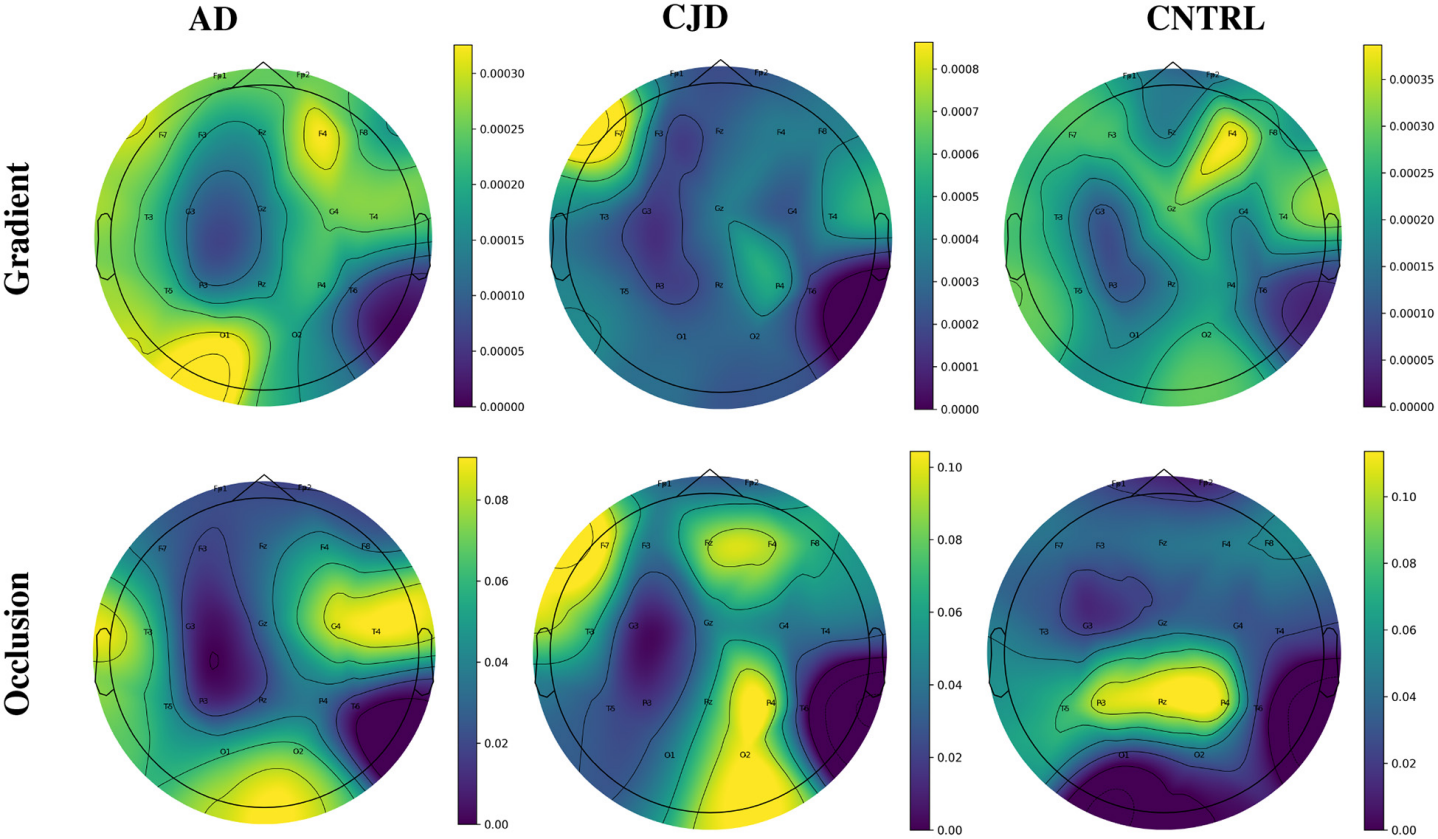
Global explanations of EEG trials. The **top** row shows gradient-based explanations, while the **bottom** row shows occlusion-based explanations across AD, CJD, and CNTRL subjects.

Figure 8 presents the global explanations for all subjects in each class (AD, CJD, and CNTRL), visualized as topographic maps. The spatio-temporal saliency maps S obtained for all EEG trials are first averaged, followed by temporal averaging to derive channel-wise importance scores. These scores are then projected onto a scalp layout to highlight the spatial distribution of discriminative neural activity. The resulting topomaps reveal distinct spatial patterns across the classes, indicating class-specific channel importance. It can be observed from the global explanations for AD that both gradient- and occlusion-based methods agree over temporal, occipital, and partially frontal regions. For CJD, both methods fully agree on the frontal region and partially on parietal regions. For CNTRL, the agreement is low, and it can be observed in frontal, parietal, and occipital regions. In CNTRL, the reason for low agreement could be due to the fact that normal subjects have alpha rhythms in occipital and parietal regions that the occlusion-based method has captured, and sometimes increased activity is observed in frontal regions that the gradient-based method has captured. Thus, the low agreement in the CNTRL class is justified since normal subjects do not show a consistent pattern like AD and CJD.

**Figure 9:**
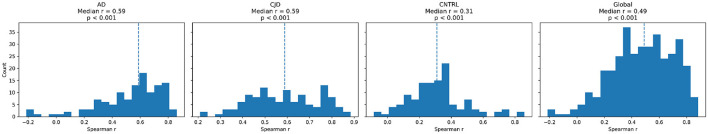
Explanation agreement. Distribution of trial-wise Spearman correlations between gradient-based and occlusion-based channel-importance for the AD, CJD, and CNTRL groups, together with the global distribution across all trials. Each subplot reports the median correlation coefficient (*r*) and the corresponding Wilcoxon signed-rank test *p*-value assessing whether the agreement is significantly greater than zero. The dashed vertical line indicates the median correlation within each distribution. Higher correlation values indicate greater agreement between the two explanation methods regarding the relative importance of EEG channels.

Since we observed some differences in highlighted regions in local explanations at the trial level by the gradient-based and occlusion-based explanation methods, To quantify the consistency between both methods, we have performed an explanation agreement analysis for all trials of all subjects. In this analysis, we have computed the Spearman correlation between gradient-based and occlusion-based channel importance across EEG channels for all subjects along-with a Wilcoxon signed-rank test *p*-value assessing whether the agreement is significant greater than zero. Figure 9 shows the distribution of Spearman correlation between gradient-based and occlusion-based channel importance across EEG channels for all subjects. The first three subplots present class-wise correlations for AD, CJD, and CNTRL, respectively, while the fourth subplot shows the global distribution across all classes. In all subplots, the sub-title shows Spearman median correlation *r* and Wilcoxon signed-rank test *p*-value, the x-axes show Spearman correlation *r*, and the y-axes show the count of trials for each value of correlation. The vertical dashed line serves as a reference for moderate agreement. The count of trials provides insight into how many trials the same channels were important in both explanation methods.

As shown in Figure 9, a significant positive agreement between the two explanation methods was observed in all groups (AD: median *r* = 0.588, *p* < 10^−20^; CJD: median *r* = 0.589, *p* < 10^−20^; CNTRL: median *r* = 0.310, *p* < 10^−20^), indicating that gradient-based and occlusion-based explanations consistently identified similar EEG channel contributions. However, the magnitude of agreement differed across groups. A Kruskal–Wallis test revealed a significant group effect (*H* = 113.495, *p* = 2.26 × 10^−25^). *Post-hoc* Mann–Whitney *U* tests demonstrated significantly lower agreement in the CNTRL group compared with both AD (*p* = 3.56 × 10^−15^) and CJD (*p* = 5.86 × 10^−25^), whereas no significant difference was observed between AD and CJD (*p* = 0.162). These results suggest that the two explanation methods capture highly consistent disease-related EEG patterns in AD and CJD. In contrast, the lower cross-method agreement observed in CNTRL may reflect the greater heterogeneity and reduced disease-specific structure of healthy-control EEG activity, leading gradient-based and occlusion-based approaches to emphasize partially different channel contributions.

In conclusion, higher correlations are observed for pathological classes (AD and CJD), indicating that both explanation methods consistently identify disease-relevant channels, whereas lower correlations in CNTRL reflect more diffuse and less discriminative EEG patterns.

## 5 Discussion

In this study, we proposed a novel interpretable framework, *EEGDecoder-x*, for decoding EEG signals from subjects with AD, CJD, and CNTRL, while simultaneously providing insights into the model's learned representations. The framework integrates a hybrid attention-based decoding model, EEGDecoder-Net, with an explainability module, EEGDecoder-XAI, enabling both high classification performance and transparent interpretation of spatio-temporal neural patterns.

The experimental evaluation, conducted using a rigorous LOSO paradigm, demonstrated that EEGDecoder-Net achieves strong classification performance, with an accuracy of 97.22 and consistently high precision, recall, F1-score compared to established baselines, including EEGNet, DeepConvNet, TCN, CTNet, and EEGConformer, confirming its effectiveness in capturing discriminative EEG features.

This superior performance is particularly relevant in the context of differential diagnosis, where distinguishing early-stage CJD from “mimics” like AD is often hampered by non-specific clinical presentations and the absence of pathognomonic EEG markers, such as periodic sharp wave complexes, which typically emerge only in later stages (30). The ability of our model to maintain high discriminative power in the absence of these terminal patterns suggests that it captures subtle, early-stage micro-alterations in neural synchrony that elude traditional visual inspection. All EEG recordings from patients with CJD were obtained during the initial diagnostic work-up, when the differential diagnosis with other neurodegenerative conditions, including AD, was still under clinical evaluation. Included EEG showed abnormalities consisting exclusively of diffuse background slowing, without periodic sharp wave complexes (PSWC) at the time of recording. This electrophysiological profile suggests a non-advanced stage of disease in terms of EEG manifestations, in which classical periodic abnormalities may not yet have emerged and EEG findings remain non-specific. In this context, the study cohort likely reflects a clinically relevant diagnostic window rather than advanced CJD with fully established EEG hallmarks.

From a signal processing perspective, PSD analysis reveals class-specific spectral differences in neural oscillations. CWT-based time—frequency analysis shows that CJD exhibits transient broadband activity, while AD shows a shift toward lower-frequency bands, consistent with cortical slowing. Despite inter-subject variability, these patterns remain observable, supporting the reliability of the processed EEG signals. These findings confirm that the model identifies neurobiologically grounded features rather than spurious correlations, a fundamental requirement for clinical meaningfulness. For AI predictions to be useful in a diagnostic setting, they must move beyond abstract scores and provide “evidence-based” interpretability. By highlighting specific “graphoelements” and rhythmic alterations, *such as focal slowing or transient discharges*, that neurologists are trained to recognize, EEGDecoder-x supports diagnostic decision-making through concordant evidence (31).

A key contribution of this work lies in the interpretability of the proposed framework, addressing the critical “black-box” problem that often prevents the clinical adoption of deep learning. Current XAI methods often lack clinical utility if they do not offer contrastive explanations–showing not just where the model looked, but why a pattern distinguishes CJD from AD (32). The EEGDecoder-XAI module provides both local and global explanations using gradient-based and occlusion-based methods. Notably, both methods consistently highlight similar temporal regions and channels for pathological classes. For AD, salient activity is observed around 1 s and between 3 and 4 s, primarily in frontal and occipital regions (e.g., F4, F8, O2), while for CJD, both methods emphasize frontal and partially temporal regions. This cross-method agreement is essential for building trust in AI-based disease detection, as it mitigates the risk of the “Clever Hans” effect, ensuring the system relies on neural signatures rather than recording artifacts (33).

Global explanations further reinforce these findings. The aggregated topographic maps reveal class-specific spatial distributions of neural activity, with strong agreement between explanation methods in AD and CJD, particularly in frontal, temporal, and occipital regions. In contrast, lower agreement is observed for the CNTRL class, which can be attributed to the inherently more variable and less discriminative nature of normal EEG patterns. For instance, occipital alpha rhythms and occasional frontal activity are captured differently by the two methods, reflecting the absence of consistent pathological signatures. The alignment between these automated explanations and the known neurophysiological characteristics of AD and CJD provides strong evidence for the clinical relevance of the framework. To further enhance trust, future iterations should provide longitudinal explanations, showing how these EEG signatures evolve with disease progression, thereby offering deeper support for prognosis and patient management.

The explainability analyses consistently highlighted frontal and occipital regions as important for classification. These patterns should be interpreted as model-derived discriminative features rather than direct localization of EEG biomarkers. In AD, the prominence of occipital electrodes is consistent with posterior cortical dysfunction and reduced posterior alpha activity reported in the literature. In CJD, although periodic sharp-wave complexes are characteristic findings, they often occur within diffuse cortical dysfunction. Since the model was trained on resting-state EEG segments rather than annotated pathological events, the highlighted regions likely reflect distributed disease-related patterns that contribute to classification.

To quantify the consistency between explanation methods, an agreement analysis based on Spearman correlation was performed across all trials and subjects along-with a Wilcoxon signed-rank test *p*-value that whether the agreement is significantly greater than zero. A significant positive agreement between the two explanation methods was observed in all groups, indicating that gradient-based and occlusion-based explanations consistently identified similar EEG channel contributions. However, the magnitude of agreement differed across groups. A Kruskal–Wallis test revealed a significant group effect, and *post-hoc* Mann–Whitney *U* tests demonstrated significantly lower agreement in the CNTRL group compared with both AD and CJD, whereas no significant difference was observed between AD and CJD. This suggests that both explanation methods reliably identify disease-relevant channels, whereas the variability in CNTRL reflects diffuse neural activity rather than model inconsistency and reflecting the inherently more variable and less discriminative nature of normal EEG patterns in the absence of a stereotyped pathological “fingerprint.” The alignment between these automated explanations and the known neurophysiological characteristics of AD and CJD provides strong evidence for the clinical relevance of the framework, suggesting that the model has effectively “learned” the spatial signature of cortical involvement in these diseases.

## 6 Conclusion

Our results suggest that quantitative EEG analysis, powered by explainable deep learning, can provide a non-invasive, cost-effective, and highly accurate alternative to more invasive biomarkers. The alignment between model explanations and known neurophysiological characteristics of AD and CJD provides strong evidence for the clinical relevance of the proposed framework. The ability of EEGDecoder-x to not only achieve high classification performance but also to offer interpretable and physiologically meaningful insights represents a significant step toward trustworthy AI in neurodegenerative disease analysis. While the current study is limited by the sample size of 36 subjects, the robustness of the LOSO validation indicates high potential for clinical translation. Future work will focus on expanding the framework to include other rapidly progressive dementias and autoimmune encephalopathies, further refining the utility of EEGDecoder-x as a decision-support tool in real-world clinical environments.

## Data Availability

The original contributions presented in the study are included in the article/Supplementary material, further inquiries can be directed to the corresponding authors.

## Footnotes

1
https://github.com/AI-Lab-UniRC/FAIR-NAEL-Database/tree/main/EEG

2
https://github.com/AI-Lab-UniRC/FAIR-NAEL-Database/releases/tag/EEG_AD_NAEL

3
https://braindecode.org/stable/index.html

## References

[1] Uyanik H Sengur A Salvi M Tan RS Tan JH Acharya UR. Automated detection of neurological and mental health disorders using EEG signals and artificial intelligence: a systematic review. WIRes Data Mining Knowl Discov. (2025) 15:e70002. doi: 10.1002/widm.70002

[2] Moraveji M Mansouri N. Recent advances in computational and machine-learning approaches for Alzheimer's disease classification: a comprehensive review. Arch Comput Methods Eng. (2025) 33:1–64. doi: 10.1007/s11831-025-10476-5

[3] Lyu R. Deep learning approaches for EEG-based healthcare applications: a comprehensive review. Front Hum Neurosci. (2026) 19:1689073. doi: 10.3389/fnhum.2025.1689073 41657359 PMC12876152

[4] Niedermeyer E da Silva FL. Electroencephalography: Basic Principles, Clinical Applications, and Related Fields. Philadelphia, PN: Lippincott Williams & Wilkins (2005).

[5] Jeong J. EEG dynamics in patients with Alzheimer's disease. Clin Neurophysiol. (2004) 115:1490–505. doi: 10.1016/j.clinph.2004.01.001 15203050

[6] Fatourechi M Bashashati A Ward RK Birch GE. EMG and EOG artifacts in brain computer interface systems: a survey. Clin Neurophysiol. (2007) 118:480–94. doi: 10.1016/j.clinph.2006.10.019 17169606

[7] Lotte F Bougrain L Cichocki A Clerc M Congedo M Rakotomamonjy A . A review of classification algorithms for EEG-based brain-computer interfaces. J Neural Eng. (2018) 15:031005. doi: 10.1088/1741-2552/aab2f2 29488902

[8] Schirrmeister RT Springenberg JT Fiederer LDJ Glasstetter M Eggensperger K Tangermann M . Deep learning with convolutional neural networks for EEG decoding and visualization. Hum Brain Mapp. (2017) 38:5391–420. doi: 10.1002/hbm.23730 28782865 PMC5655781

[9] Suffian M Ieracitano C Morabito FC Mammone N. An explainable 3D-deep learning model for EEG decoding in brain-computer interface applications. Int J Neural Syst. (2025) 35:2550073. doi: 10.1142/S012906572550073X 41109958

[10] Mouazen B Bendaouia A Bellakhdar O Laghdaf K Ennair A Abdelwahed EH . Transparent EEG analysis: leveraging Autoencoders, Bi-LSTMs, and SHAP for improved neurodegenerative diseases detection. Sensors. (2025) 25:5690. doi: 10.3390/s25185690 41012929 PMC12473364

[11] Li Z Wang H Li L. Task-related EEG as a biomarker for preclinical Alzheimer's disease: an explainable deep learning approach. Biomimetics. (2025) 10:468. doi: 10.3390/biomimetics10070468 40710281 PMC12292204

[12] Dauwels J Vialatte F Cichocki A. Diagnosis of Alzheimer's disease from EEG signals: where are we standing? Curr Alzheimer Res. (2010) 7:487–505. doi: 10.2174/156720510792231720 20455865

[13] Mammone N Bonanno L Salvo SD Marino S Bramanti P Bramanti A . Permutation disalignment index as an indirect, EEG-based, measure of brain connectivity in MCI and AD patients. Int J Neural Syst. (2017) 27:1750020. doi: 10.1142/S0129065717500204 28355927

[14] Mammone N Ieracitano C Adeli H Bramanti A Morabito FC. Permutation Jaccard distance-based hierarchical clustering to estimate EEG network density modifications in MCI subjects. IEEE Trans Neural Netw Learn Syst. (2018) 29:5122–35. doi: 10.1109/TNNLS.2018.2791644 29994428

[15] Amezquita-Sanchez JP Mammone N Morabito FC Marino S Adeli H. A novel methodology for automated differential diagnosis of mild cognitive impairment and the Alzheimer's disease using EEG signals. J Neurosci Methods. (2019) 322:88–95. doi: 10.1016/j.jneumeth.2019.04.013 31055026

[16] Lawhern VJ Solon AJ Waytowich NR Gordon SM Hung CP Lance BJ. EEGNet: a compact convolutional neural network for EEG-based brain-computer interfaces. J Neural Eng. (2018) 15. doi: 10.1088/1741-2552/aace8c 29932424

[17] Roy Y Banville H Albuquerque I Gramfort A Falk TH Faubert J. Deep learning-based electroencephalography analysis: a systematic review. J Neural Eng. (2019) 16:051001. doi: 10.1088/1741-2552/ab260c 31151119

[18] Morabito FC Campolo M Mammone N Versaci M Franceschetti S Tagliavini F . Deep learning representation from electroencephalography of early-stage Creutzfeldt-Jakob disease and features for differentiation from rapidly progressive dementia. Int J Neural Syst. (2017) 27:1650039. doi: 10.1142/S0129065716500398 27440465

[19] Bai S Kolter JZ Koltun V. An empirical evaluation of generic convolutional and recurrent networks for sequence modeling. arXiv. [preprint]. (2018). arXiv:1803.01271. doi: 10.48550/arXiv.1803.01271

[20] Vaswani A Shazeer N Parmar N Uszkoreit J Jones L Gomez AN . Attention is all you need. In: Advances in neural information processing systems (Red Hook) (2017). p. 6000–6010.

[21] Wang Y Mammone N Petrovsky D Tzallas AT Morabito FC Zhang X. Adformer: a multi-granularity transformer for EEG-based alzheimer's disease assessment. arXiv. [preprint]. (2024). arXiv:2409.00032. doi: 10.48550/arXiv.2409.00032

[22] Zhao W Jiang X Zhang B Xiao S Weng S. CTNet: a convolutional transformer network for EEG-based motor imagery classification. Sci Rep. (2024) 14:20237. doi: 10.1038/s41598-024-71118-7 39215126 PMC11364810

[23] Song Y Zheng Q Liu B Gao X. EEG conformer: convolutional transformer for EEG decoding and visualization. IEEE Trans Neural Syst Rehabil Eng. (2022) 31:710–9. doi: 10.1109/TNSRE.2022.3230250 37015413

[24] Lopes M Cassani R Falk TH. Using CNN saliency maps and EEG modulation spectra for improved and more interpretable machine learning-based Alzheimer's disease diagnosis. Comput Intell Neurosci. (2023) 2023:3198066. doi: 10.1155/2023/3198066 36818579 PMC9931465

[25] Morabito FC Ieracitano C Mammone N. An explainable Artificial Intelligence approach to study MCI to AD conversion via HD-EEG processing. Clin EEG Neurosci. (2023) 54:51–60. doi: 10.1177/15500594211063662 34889152

[26] Bunterngchit C Baniata LH Rasool A. NeuroXAI: explainable deep learning for EEG-based detection of Alzheimer's and Parkinson's diseases. In: 2025 IEEE 25th international conference on bioinformatics and bioengineering (BIBE). Athens: IEEE (2025). p. 612–9. doi: 10.1109/BIBE66822.2025.00107

[27] Jack CR Jr Bennett DA Blennow K Carrillo MC Dunn B Haeberlein SB . NIA-AA research framework: toward a biological definition of Alzheimer's disease. Alzheimers Dement. (2018) 14:535–62. doi: 10.1016/j.jalz.2018.02.018 29653606 PMC5958625

[28] Dubois B Villain N Frisoni GB Rabinovici GD Sabbagh M Cappa S . Clinical diagnosis of Alzheimer's disease: recommendations of the International Working Group. Lancet Neurol. (2021) 20:484–96. doi: 10.1016/S1474-4422(21)00066-1 33933186 PMC8339877

[29] Hermann P Appleby B Brandel JP Caughey B Collins S Geschwind MD . Biomarkers and diagnostic guidelines for sporadic Creutzfeldt-Jakob disease. Lancet Neurol. (2021) 20:235–46. doi: 10.1016/S1474-4422(20)30477-4 33609480 PMC8285036

[30] Zerr I Kallenberg K Summers D Romero C Taratuto A Heinemann U . Updated clinical diagnostic criteria for sporadic Creutzfeldt-Jakob disease. Brain. (2009) 132:2659–68. doi: 10.1093/brain/awp191 19773352 PMC2759336

[31] Kelly CJ Karthikesalingam A Suleyman M Corrado G King D. Key challenges for delivering clinical impact with artificial intelligence. BMC Med. (2019) 17:195. doi: 10.1186/s12916-019-1426-2 31665002 PMC6821018

[32] Miller T. Explanation in artificial intelligence: insights from the social sciences. Artif Intell. (2019) 267:1–38. doi: 10.1016/j.artint.2018.07.007

[33] Lapuschkin S Wäldchen S Binder A Montavon G Samek W Müller KR. Unmasking Clever Hans predictors and assessing what machines really learn. Nat Commun. (2019) 10:1096. doi: 10.1038/s41467-019-08987-4 30858366 PMC6411769

