# Supplementary material for: EEGDecoder-x: an explainable deep learning framework for cross-subject EEG-based detection of Alzheimer's and Creutzfeldt–Jakob disease
Source: Front Neurol. 2026 Jul 20;17:1851752. doi: 10.3389/fneur.2026.1851752 (PMC13429416; doi:10.3389/fneur.2026.1851752)
Supplement: Supplementary file 1 [file Data_Sheet_1.pdf]

(Supplementary Material) EEGDecoder-x: An Explainable Deep Learning Framework for Cross-Subject EEG-Based Detection of Alzheimer's and Creutzfeldt–Jakob Disease

SUPPLEMENTARY RESULTS

List of Supplementary Materials.

Supplementary Table S1. Complete subject-level LOSO performance comparison across all models.

Supplementary Figure S1. Subject-level confusion matrices for all models.

Supplementary Figure S2. Local explanations for different subjects of Alzheimer Disease (AD).

Supplementary Figure S3. Local explanations for different subjects of Creutzfeldt-Jakob Disease (CJD).

Supplementary Figure S4. Local explanations for different subjects of healthy control (CNTRL).

Table S1. Complete subject-level LOSO performance comparison across all models.

	EEGNet				EEGConformer				DeepConvNet				CTNet				TCN				EEGDecoder-Net (Proposed)			
Subject	Acc (%)	Prec (%)	Rec (%)	F1 (%)	Acc (%)	Prec (%)	Rec (%)	F1 (%)	Acc (%)	Prec (%)	Rec (%)	F1 (%)	Acc (%)	Prec (%)	Rec (%)	F1 (%)	Acc (%)	Prec (%)	Rec (%)	F1 (%)	Acc (%)	Prec (%)	Rec (%)	F1 (%)
AD_010	25.0	33.3	8.3	13.3	7.5	33.3	2.5	4.7	0.0	0.0	0.0	0.0	0.0	0.0	0.0	0.0	2.5	33.3	0.8	1.6	0.0	0.0	0.0	0.0
AD_008	100	100	100	100	100	100	100	100	100	100	100	100	100	100	100	100	100	100	100	100	100	100	100	100
AD_000	100	100	100	100	100	100	100	100	97.5	50.0	48.8	49.4	100	100	100	100	100	100	100	100	100	100	100	100
AD_001	20.0	50.0	10.0	16.7	67.5	50.0	33.8	40.3	52.5	50.0	26.3	34.4	70.0	50.0	35.0	41.2	82.5	50.0	41.3	45.2	100	100	100	100
AD_002	90.0	50.0	45.0	47.4	100	100	100	100	100	100	100	100	82.5	50.0	41.3	45.2	100	100	100	100	100	100	100	100
AD_005	100	100	100	100	100	100	100	100	100	100	100	100	100	100	100	100	100	100	100	100	100	100	100	100
AD_011	97.5	50.0	48.8	49.4	100	100	100	100	97.5	50.0	48.8	49.4	87.5	50.0	43.8	46.7	100	100	100	100	100	100	100	100
AD_006	100	100	100	100	100	100	100	100	90.0	50.0	45.0	47.4	95.0	50.0	47.5	48.7	100	100	100	100	100	100	100	100
AD_009	100	100	100	100	100	100	100	100	100	100	100	100	100	100	100	100	100	100	100	100	100	100	100	100
AD_007	100	100	100	100	97.5	50.0	48.8	49.4	55.0	50.0	27.5	35.5	77.5	33.3	25.8	29.1	100	100	100	100	100	100	100	100
AD_003	97.5	50.0	48.8	49.4	100	100	100	100	100	100	100	100	100	100	100	100	100	100	100	100	100	100	100	100
AD_004	100	100	100	100	100	100	100	100	100	100	100	100	100	100	100	100	100	100	100	100	100	100	100	100
CJD_000	100	100	100	100	100	100	100	100	100	100	100	100	100	100	100	100	100	100	100	100	100	100	100	100
CJD_005	100	100	100	100	100	100	100	100	100	100	100	100	100	100	100	100	100	100	100	100	100	100	100	100
CJD_004	100	100	100	100	97.5	50.0	48.8	49.4	100	100	100	100	97.5	50.0	48.8	49.4	100	100	100	100	100	100	100	100
CJD_007	92.5	33.3	30.8	32.0	100	100	100	100	100	100	100	100	100	100	100	100	100	100	100	100	100	100	100	100
CJD_003	97.5	50.0	48.8	49.4	100	100	100	100	92.5	50.0	46.3	48.1	95.0	50.0	47.5	48.7	100	100	100	100	95.0	50.0	47.5	48.7
CJD_009	100	100	100	100	100	100	100	100	100	100	100	100	100	100	100	100	100	100	100	100	100	100	100	100
CJD_011	100	100	100	100	100	100	100	100	90.0	50.0	45.0	47.4	100	100	100	100	100	100	100	100	100	100	100	100
CJD_010	97.5	50.0	48.8	49.4	100	100	100	100	100	100	100	100	97.5	50.0	48.8	49.4	100	100	100	100	100	100	100	100
CJD_006	100	100	100	100	100	100	100	100	100	100	100	100	100	100	100	100	100	100	100	100	100	100	100	100
CJD_008	100	100	100	100	100	100	100	100	100	100	100	100	100	100	100	100	100	100	100	100	100	100	100	100
CJD_001	100	100	100	100	100	100	100	100	100	100	100	100	100	100	100	100	100	100	100	100	100	100	100	100
CJD_002	97.5	50.0	48.8	49.4	100	100	100	100	95.0	50.0	47.5	48.7	100	100	100	100	100	100	100	100	100	100	100	100
CNTRL_003	100	100	100	100	100	100	100	100	100	100	100	100	100	100	100	100	100	100	100	100	100	100	100	100
CNTRL_006	100	100	100	100	100	100	100	100	100	100	100	100	100	100	100	100	100	100	100	100	100	100	100	100
CNTRL_002	100	100	100	100	100	100	100	100	100	100	100	100	100	100	100	100	100	100	100	100	100	100	100	100
CNTRL_001	100	100	100	100	100	100	100	100	100	100	100	100	100	100	100	100	100	100	100	100	100	100	100	100
CNTRL_010	100	100	100	100	100	100	100	100	100	100	100	100	100	100	100	100	100	100	100	100	100	100	100	100
CNTRL_011	100	100	100	100	100	100	100	100	100	100	100	100	100	100	100	100	100	100	100	100	100	100	100	100
CNTRL_008	100	100	100	100	100	100	100	100	100	100	100	100	100	100	100	100	100	100	100	100	100	100	100	100
CNTRL_009	100	100	100	100	100	100	100	100	100	100	100	100	100	100	100	100	100	100	100	100	100	100	100	100
CNTRL_007	100	100	100	100	100	100	100	100	100	100	100	100	100	100	100	100	100	100	100	100	100	100	100	100
CNTRL_004	100	100	100	100	100	100	100	100	100	100	100	100	100	100	100	100	100	100	100	100	100	100	100	100
CNTRL_005	100	100	100	100	100	100	100	100	100	100	100	100	100	100	100	100	100	100	100	100	100	100	100	100
CNTRL_000	100	100	100	100	100	100	100	100	100	100	100	100	100	100	100	100	100	100	100	100	100	100	100	100
**Mean**	**94.00**	**88.00**	**85.00**	**86.00**	**94.70**	**94.88**	**94.75**	**94.67**	**93.29**	**94.00**	**93.37**	**93.28**	**94.07**	**94.38**	**94.15**	**94.00**	**93.36**	**93.51**	**93.41**	**93.30**	**97.22**	**97.16**	**97.07**	**97.01**
**Std**	**17.69**	**23.51**	**28.22**	**22.41**	**15.95**	**17.19**	**21.84**	**18.62**	**19.08**	**25.31**	**28.27**	**24.11**	**17.36**	**26.10**	**28.05**	**23.92**	**16.20**	**13.51**	**18.70**	**17.15**	**15.43**	**18.16**	**18.34**	**17.90**

Subject-level statistical comparison of model performance. To complement the aggregate performance metrics reported in the main manuscript, statistical analyses were performed using the subject-wise LOSO classification results summarized in Table S1. Because all models were evaluated on the same 36 subjects, the resulting measurements constitute paired observations and are suitable for repeated-measures statistical testing.

A Friedman test was first applied across the six evaluated architectures (EEGNet, EEGConformer, DeepConvNet, CTNet, TCN, and EEGDecoder-Net). The analysis revealed a significant overall difference among models ($\chi^2 = 23.49$, $p = 2.72 \times 10^{-4}$), confirming that model architecture significantly influenced classification performance at the subject level.

To identify which models differed from the proposed approach, pairwise Wilcoxon signed-rank tests were subsequently conducted between EEGDecoder-Net and each baseline. Significant improvements were observed relative to DeepConvNet ($p = 0.0115$) and CTNet ($p = 0.0115$). In contrast, differences relative to EEGNet ($p = 0.0803$), EEGConformer ($p = 0.4615$), and TCN ($p = 0.6547$) were not statistically significant.

The modest magnitude of several pairwise differences is attributable to a pronounced ceiling effect in the dataset. Specifically, many subjects achieved perfect classification performance across multiple architectures, resulting in highly compressed score distributions near 100% accuracy. Consequently, although the proposed model achieved the highest mean performance, statistical comparisons based on subject-level outcomes indicate that its advantage is concentrated primarily over DeepConvNet and CTNet, while performance remains comparable to EEGNet, EEGConformer, and TCN. These findings provide additional evidence that the proposed model achieves robust and competitive performance across subjects under the LOSO evaluation framework.

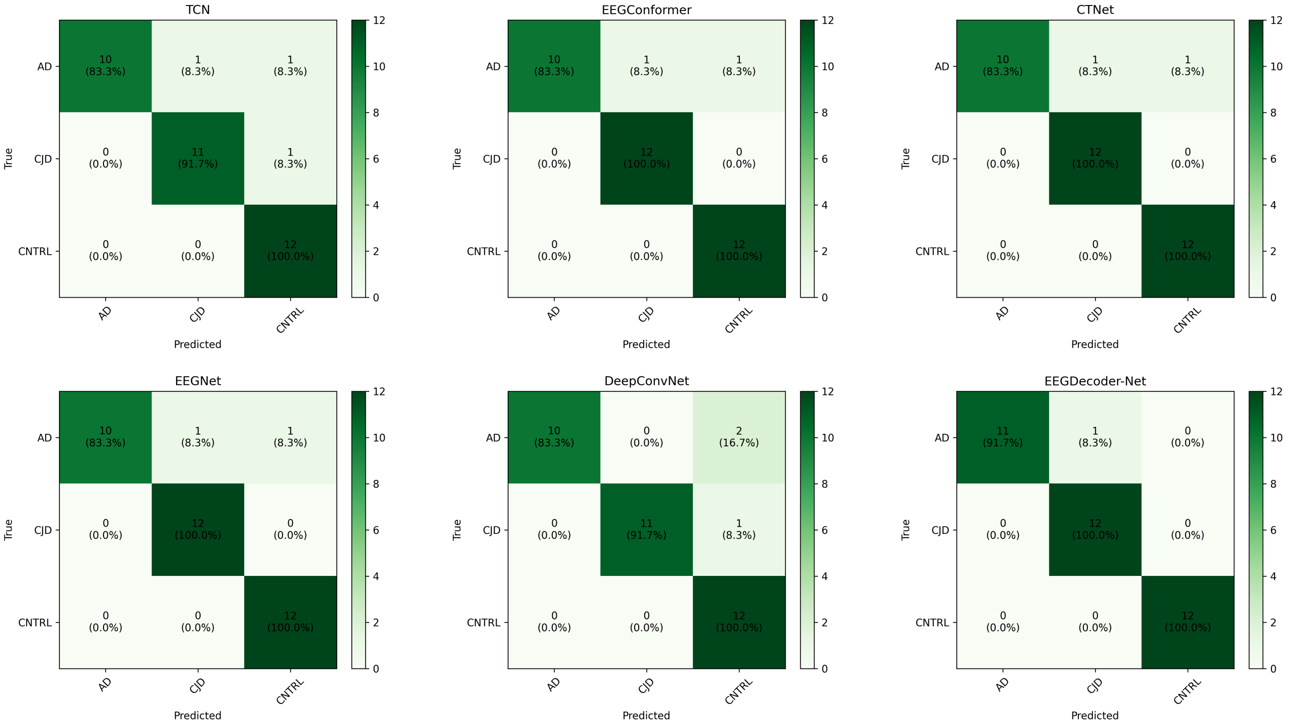

Figure S1. Confusion matrices produced by TCN, EEGConformer, CTNet, EEGNet, DeepConvNet, and EEGDecoder-Net in the AD vs CJD vs CNTRL classification using the LOSO approach. The confusion matrices show performance on subject-level.

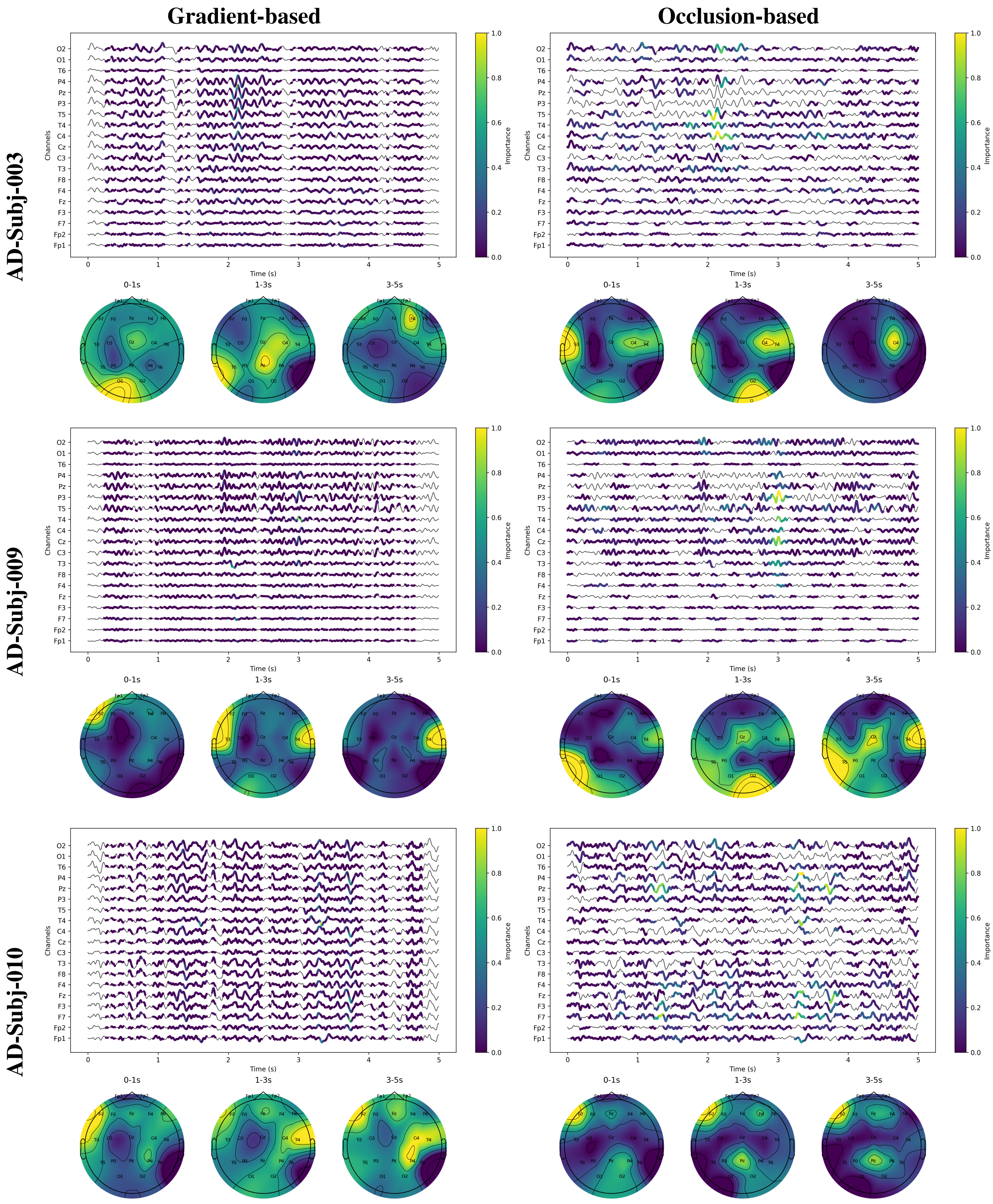

Figure S2. Local explanations are shown with a topographical map. The rows in the plot shows to different subjects of Alzheimer Disease (AD) and columns shows specific explanations strategy for these classes (gradient-based and occlusion-based explanations).

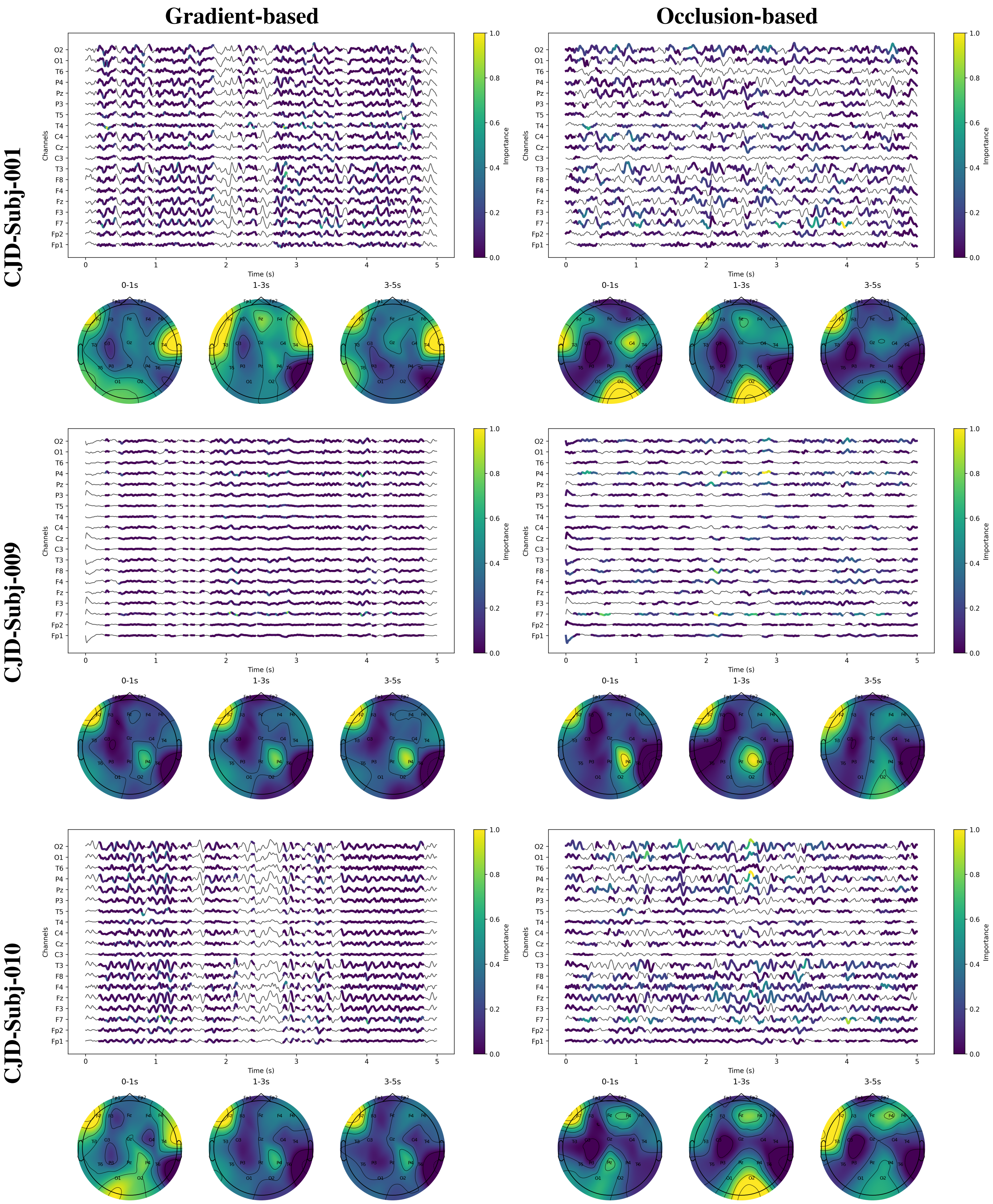

Figure S3. Local explanations are shown with a topographical map. The rows in the plot shows to different subjects of Creutzfeldt-Jakob Disease (CJD) and columns shows specific explanations strategy for these classes (gradient-based and occlusion-based explanations).

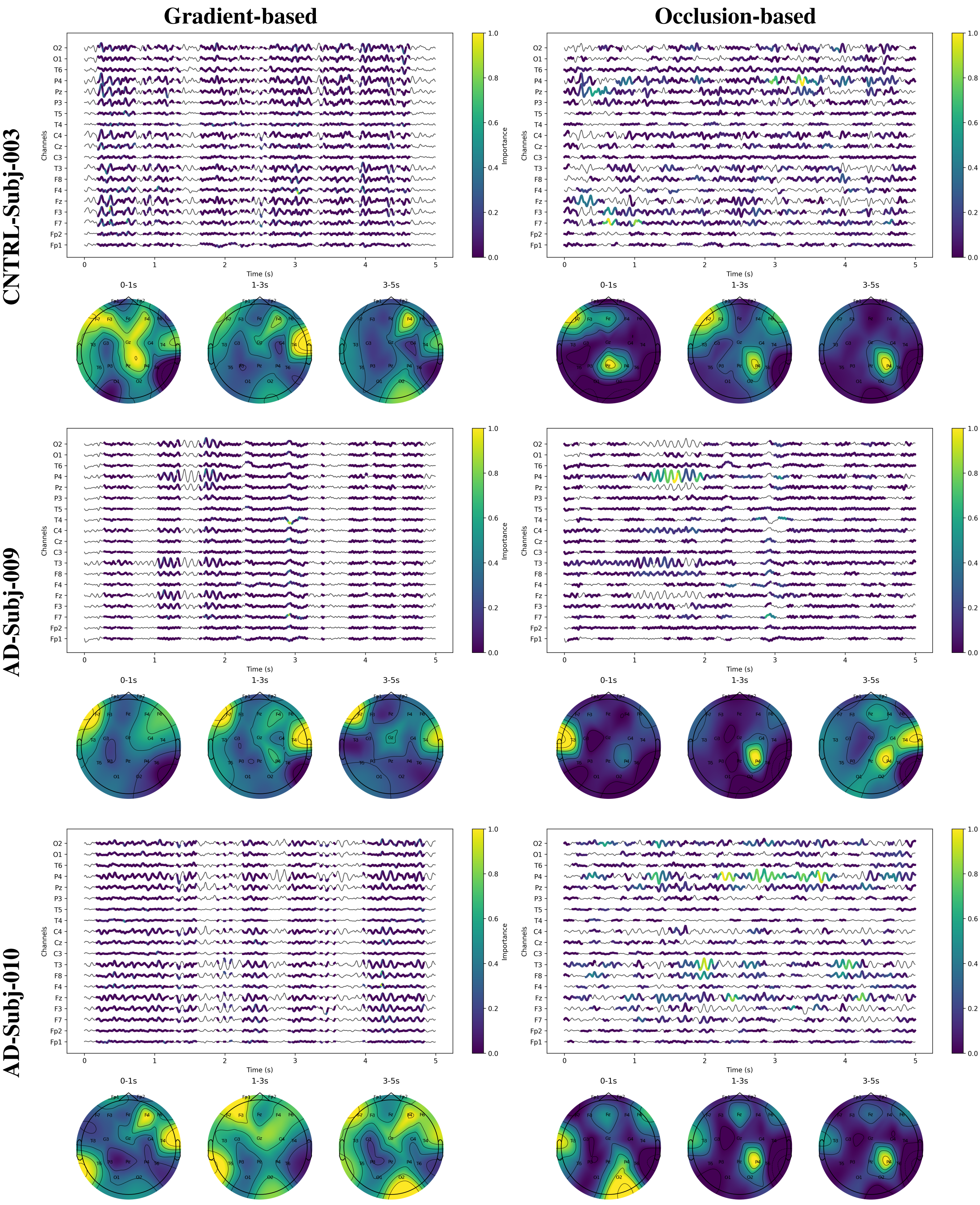

Figure S4. Local explanations are shown with a topographical map. The rows in the plot shows to different subjects of Control (CNTRL) and columns shows specific explanations strategy for these classes (gradient-based and occlusion-based explanations).
